# Pseudomonas aeruginosa Production of Hydrogen Cyanide Leads to Airborne Control of Staphylococcus aureus Growth in Biofilm and *In Vivo* Lung Environments

**DOI:** 10.1128/mbio.02154-22

**Published:** 2022-09-21

**Authors:** Sylvie Létoffé, Yongzheng Wu, Sophie E. Darch, Christophe Beloin, Marvin Whiteley, Lhousseine Touqui, Jean-Marc Ghigo

**Affiliations:** a Institut Pasteur, Université de Paris Cité, CNRS UMR 6047, Genetics of Biofilms Laboratory, Paris, France; b Institut Pasteur, Université de Paris Cité, CNRS UMR3691, Cellular Biology of Microbial Infection Laboratory, Paris, France; c Department of Molecular Medicine, University of South Florida, Tampa, Florida, USA; d School of Biological Sciences, Georgia Institute of Technology, Atlanta, Georgia, USA; e Institut Pasteur, Université de Paris Cité, Mucoviscidose et Bronchopathies Chroniques, Paris, France; f Centre de Recherche Saint-Antoine, Sorbonne Université, Inserm, Paris, France; University of Geneva

**Keywords:** volatile compounds, *Staphylococcus aureus*, *Pseudomonas aeruginosa*, bacterial coinfection, hydrogen cyanide

## Abstract

Diverse bacterial volatile compounds alter bacterial stress responses and physiology, but their contribution to population dynamics in polymicrobial communities is not well known. In this study, we showed that airborne volatile hydrogen cyanide (HCN) produced by a wide range of Pseudomonas aeruginosa clinical strains leads to at-a-distance *in vitro* inhibition of the growth of a wide array of Staphylococcus aureus strains. We determined that low-oxygen environments not only enhance P. aeruginosa HCN production but also increase S. aureus sensitivity to HCN, which impacts P. aeruginosa-S. aureus competition in microaerobic *in vitro* mixed biofilms as well as in an *in vitro* cystic fibrosis lung sputum medium. Consistently, we demonstrated that production of HCN by P. aeruginosa controls S. aureus growth in a mouse model of airways coinfected by P. aeruginosa and S. aureus. Our study therefore demonstrates that P. aeruginosa HCN contributes to local and distant airborne competition against S. aureus and potentially other HCN-sensitive bacteria in contexts relevant to cystic fibrosis and other polymicrobial infectious diseases.

## INTRODUCTION

Bacteria release a wide diversity of volatile molecules contributing to cross-kingdom interactions with fungi, plants, and animals (1, 2). Bacterial volatile compounds also play a role in bacterial physiology by altering stress responses, antibiotic resistance, biofilm formation, and expression of virulence factors. Although these interactions likely contribute to bacterial population dynamics, relatively little is known regarding interactions mediated by volatile compounds in polymicrobial communities (2–8). Cystic fibrosis (CF) is a common genetic disease in which the patients’ airways are often colonized by multiple bacterial pathogens, including Pseudomonas aeruginosa and Staphylococcus aureus, that are frequently found in association in the same lung lobes (9–15). Whereas S. aureus usually colonizes the airways first during CF infection, it is later outcompeted and replaced by P. aeruginosa (12, 16–20).

Several P. aeruginosa extracellular factors inhibiting S. aureus growth could contribute to this colonization shift during CF infection, including siderophores, 4-hydroxy-2-heptylquinoline-*N*-oxide (HQNO), proteases, redox compounds, and surface-active compounds (19, 21–26). In contrast, less is known about how competitive interactions of P. aeruginosa are mediated via the production of volatile compounds and their impact on the dynamics of coinfections with S. aureus (27–29).

It has long been recognized that P. aeruginosa metabolism produces volatile hydrogen cyanide (HCN), which can rapidly diffuse into the environment (30, 31). HCN is an inhibitor of cytochrome *c* oxidases and other metalloenzymes that bind iron, leading to the inhibition of the respiratory chain (30). HCN production is restricted to Pseudomonas, *Chromobacterium*, *Rhizobium*, and several cyanobacterial species that avoid autointoxication by expressing HCN-insensitive cytochrome oxidase (31). P. aeruginosa HCN is produced by the oxidative decarboxylation of glycine mediated by membrane-bound cyanide synthases encoded by the *hcnABC* operon (32–34)*. hcnABC* expression is maximal between 34°C and 37°C and transcriptionally upregulated under microaerobic conditions and conditions of high bacterial cell density (31, 35). Consistently, HCN production by P. aeruginosa is regulated by the anaerobic regulator Anr and the LasR and RhlR quorum sensing regulators (36). HCN is therefore produced under environmental conditions leading to the induction of P. aeruginosa virulence factors, including the synthesis of alginate, a constituent of P. aeruginosa biofilm matrix and a major virulence factor in the lungs of CF patients (37–39).

Considering that HCN was shown to poison a wide range of eukaryotic organisms (2, 40–42), it was hypothesized that cyanogenesis could also poison HCN-sensitive bacteria in a range of polymicrobial niches (26, 28, 30, 31, 39, 43, 44). However, whereas P. aeruginosa HCN was shown to inhibit the growth of a wide range of staphylococci, including S. aureus (45), the direct contribution of HCN to P. aeruginosa dominance over S. aureus within polymicrobial niches such as biofilms and infected lungs is still unclear.

Here, we showed that exposure to airborne HCN produced by P. aeruginosa inhibits S. aureus growth and influences the dynamics of P. aeruginosa-S. aureus interactions in *in vitro* mixed biofilms. We determined that HCN production is widespread among P. aeruginosa clinical strains and particularly active in low-oxygen (microaerobic) conditions against a representative panel of S. aureus isolates. We also demonstrated that P. aeruginosa HCN controls S. aureus growth in an *in vitro* CF lung sputum model as well as in a mouse model of airway coinfection by P. aeruginosa and S. aureus. Our study therefore shows that volatile HCN mediates local and at-a-distance growth control against S. aureus and potentially other HCN-sensitive bacteria in contexts relevant to CF and other polymicrobial infectious diseases.

## RESULTS

### Production of volatile hydrogen cyanide by P. aeruginosa leads to airborne inhibition of S. aureus growth.

**FIG 1:**
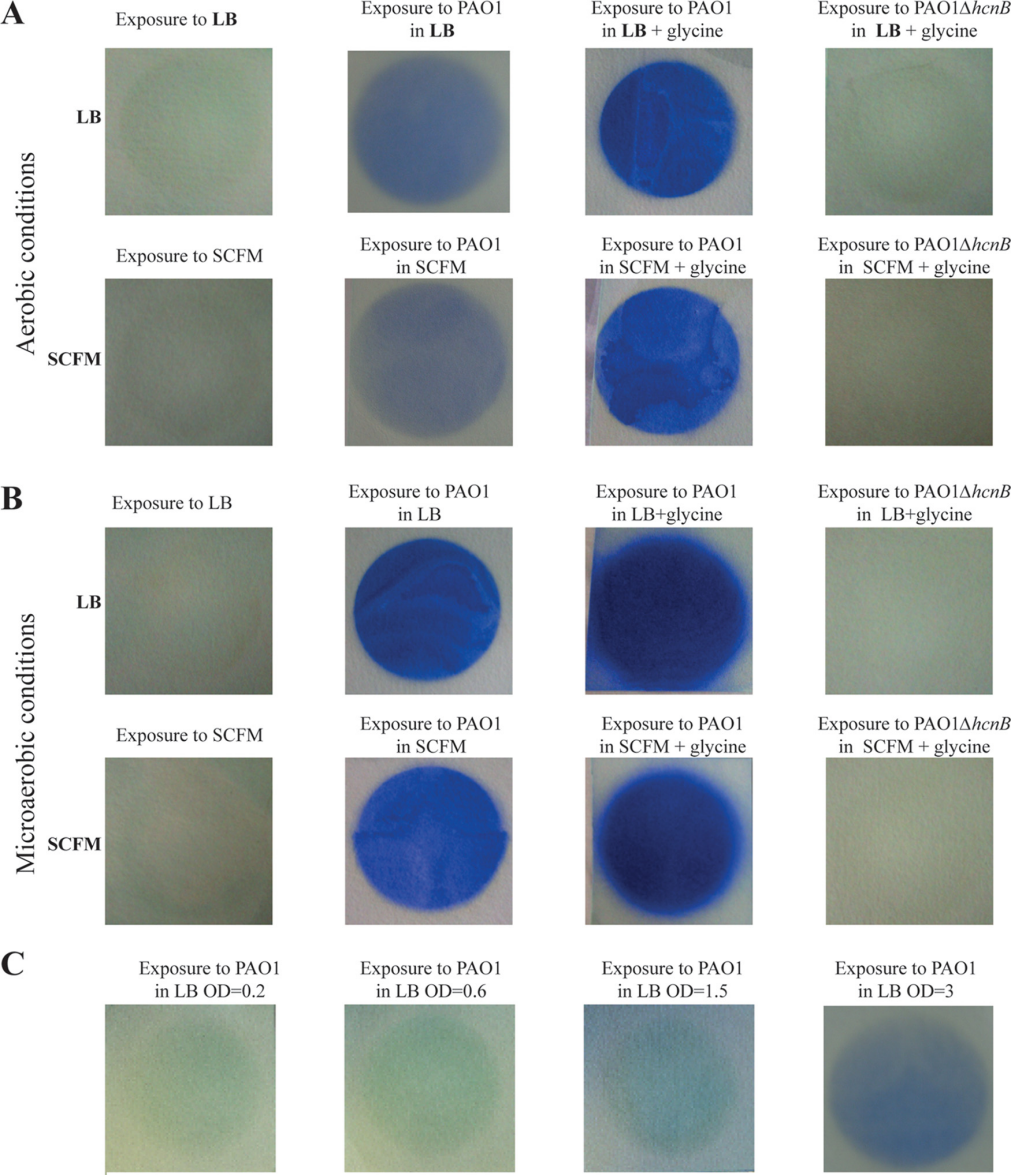
Semiquantitative detection of volatile HCN emitted from P. aeruginosa cultures under various conditions. Semiquantitative HCN detection upon HCN reaction with copper(II) ethylacetoacetate and 4,4′-methylenebis-(*N*,*N*-dimethylaniline). (A) Semiquantitative detection of volatile HCN emitted from P. aeruginosa WT and PAO1*ΔhcnB*, in LB or SCFM2 medium supplemented or not with 0.4% (wt/vol) glycine, after 24 h of incubation at 37°C under aerobic conditions. (B) Semiquantitative detection of volatile HCN from P. aeruginosa WT and PAO1*ΔhcnB*, in LB or SCFM2 medium supplemented or not with 0.4% (wt/vol) glycine, after 24 h of incubation at 37°C under microaerobic conditions. Pictures were taken after 24 h of incubation at 37°C using the 2-petri-dish assay described in Fig. S1. Each experiment was performed at least three times. (C) Semiquantitative detection of volatile HCN showed an increased production of HCN by P. aeruginosa PAO1 at different stages of growth in LB medium. Each experiment was performed at least three times.

**FIG 2:**
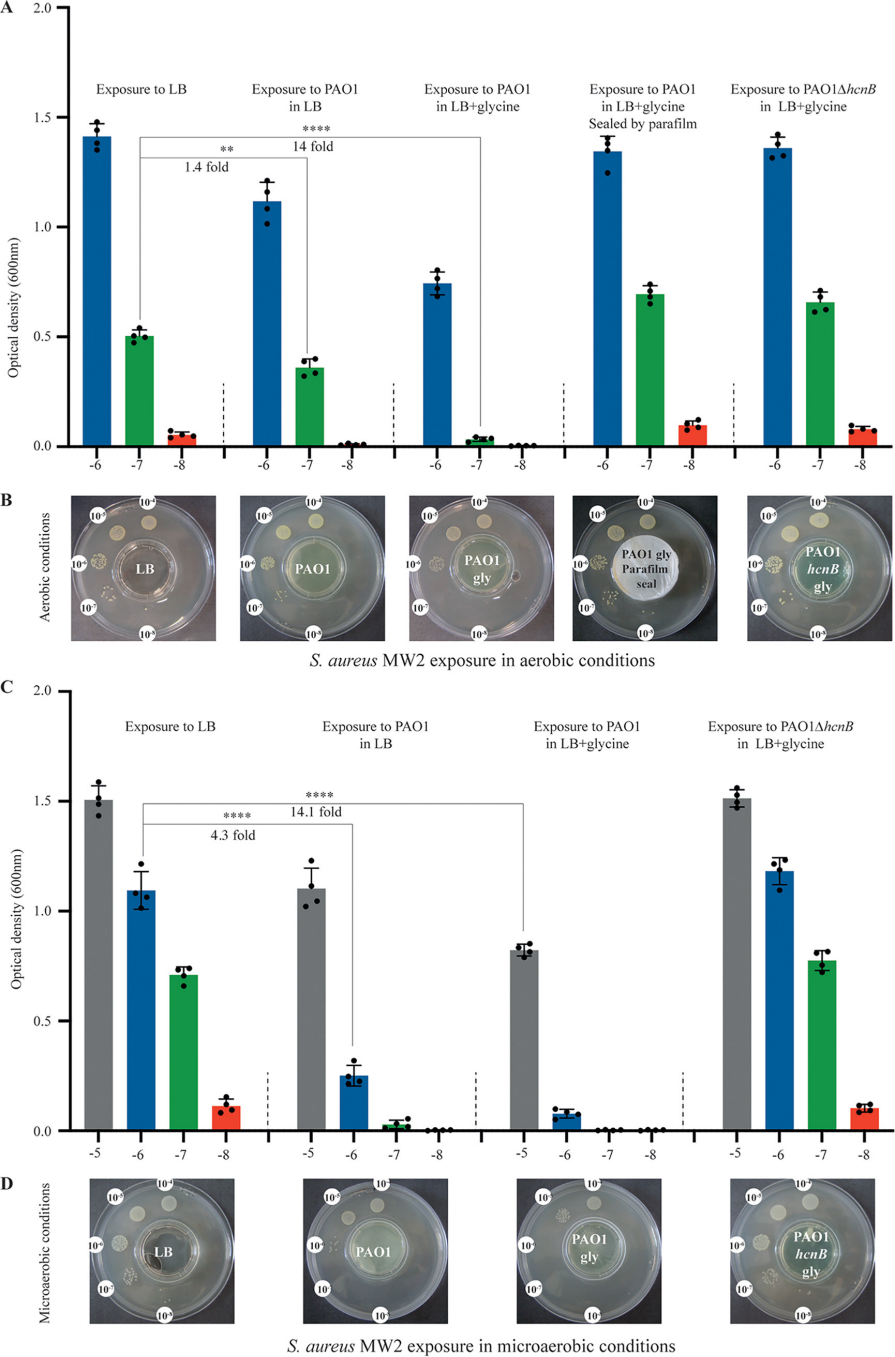
P. aeruginosa production of HCN in LB leads to airborne inhibition of S. aureus growth. (A) Quantification of the effect of exposure to P. aeruginosa HCN on S. aureus MW2 growth in LB aerobic conditions. Bacteria were grown on 10^−4^ to 10^−8^ dilution spots (see Fig. S1 for setup) and exposed to P. aeruginosa HCN or not. Each spot was punched out from the LB agar plate and resuspended in PBS, and the corresponding OD_600_ was determined. The fold differences observed between different conditions at comparable dilution were calculated based on the ratio of the means of 4 independent quantifications at each dilution. (B) Serial dilution of S. aureus MW2 exposed to P. aeruginosa WT or PAO1*ΔhcnB* cultures in LB supplemented or not with 0.4% (wt/vol) glycine in the 2-petri-dish assay (Fig. S1). No inhibition of S. aureus MW2 growth was observed when the middle plate containing P. aeruginosa culture was covered and sealed with Parafilm. Pictures were taken after 24 h of incubation at 37°C under aerobic conditions. Each experiment was performed at least three times. (C and D) As for panels A and B, except that the experiments were performed under microaerobic conditions. Statistics correspond to a two-tailed unpaired *t* test with Welch correction. **, *P* ≤ 0.01; ****, *P* ≤ 0.0001.

To determine whether HCN released by P. aeruginosa could inhibit the growth of S. aureus, we first tested HCN production by PAO1, a commonly used strain of P. aeruginosa isolated from a wound infection (46). Using a semiquantitative HCN detection method based on the intensity of blue color produced upon HCN reaction with copper(II) ethylacetoacetate and 4,4′-methylenebis-(*N*,*N*-dimethylaniline) (47) (see Fig. S1 in the supplemental material), we detected an HCN signal emitted from wild-type (WT) P. aeruginosa PAO1 grown in LB, which increased upon glycine supplementation (Fig. 1A and B). In contrast, no HCN signal could be detected from a *ΔhcnB* mutant, which lacks HCN production (Fig. 1A and B). Whereas exposure of P. aeruginosa PAO1 to volatile HCN emitted by PAO1 cultures in the setup described in Fig. S1 did not affect its own growth (Fig. S2), exposure of S. aureus MW2 reduced its growth by 1.4-fold, and this reduction was further increased by 10-fold upon PAO1 culture supplementation with glycine (Fig. 2A and B). This growth inhibition was not observed in the HCN-deficient mutant PAO1*ΔhcnB*, a phenotype that could be fully complemented upon introduction of a plasmid expressing *hcnBC* into PAO1*ΔhcnB* (Fig. S3). Finally, no inhibition of S. aureus MW2 growth could be observed when the emitting plate containing P. aeruginosa cultures were closed and sealed, confirming the contribution of volatile HCN to S. aureus MW2 growth inhibition (Fig. 2A and B).

10.1128/mbio.02154-22.1 FIG S1 Two-petri-dish assay. (A) Evaluation of volatile-mediated impact on growth between physically separated bacteria. A small lidless petri dish was placed inside a larger one, which was closed with its lid. Serial dilutions of bacteria spotted on the external LB agar ring were exposed to volatile molecule released from the culture placed in the central small petri dish. Bacterial growth was monitored after 24 h of incubation at 37°C, under aerobic or microaerobic conditions. (B) Semiquantitative HCN detection: Whatman chromatography paper soaked with HCN detection reagent was laid on the surface of the uncovered small petri dish containing bacterial liquid culture releasing volatile HCN or not, and the large petri dish was then closed and incubated for 24 h at 37°C under aerobic or microaerobic conditions. Download FIG S1, PDF file, 0.7 MB. Copyright © 2022 Létoffé et al. 2022 Létoffé et al. https://creativecommons.org/licenses/by/4.0/ This content is distributed under the terms of the Creative Commons Attribution 4.0 International license.

10.1128/mbio.02154-22.2 FIG S2 P. aeruginosa growth is not inhibited by HCN produced. (A) Control showing that HCN emitted by P. aeruginosa PAO1 inhibits S. aureus MW2 *ΔsrrAB* mutant under aerobic conditions. (Top) Graph representing the quantification of the effect of exposure of S. aureus *ΔsrrAB* to WT or HCN-deficient P. aeruginosa PAO1 in LB aerobic conditions. Bacteria were grown on 10^−6^ to 10^−8^ (blue, green, and red bars, respectively) dilution spots (see Fig. S1 for setup) and exposed to P. aeruginosa HCN or not. Each spot was punched out from the LB agar plate and resuspended in PBS, and the corresponding OD_600_ was determined. The fold differences observed between different conditions at comparable dilution are indicated. They were calculated based on the ratio of the mean of 4 independent quantifications at each dilution. (Bottom) Serial dilution of S. aureus MW2*ΔsrrAB* exposed to P. aeruginosa PAO1 WT or PAO1*ΔhcnB* cultures in LB in the 2-petri-dish assay (Fig. S1). Each experiment was performed at least four times. (B) Control showing that P. aeruginosa PAO1 growth is insensitive to its own HCN. (Top) Same as panel A, except that the graph shows the quantification of the effect of exposure of P. aeruginosa PAO1 to WT or HCN-deficient P. aeruginosa PAO1 in LB under aerobic conditions. (Bottom) Serial dilution of P. aeruginosa PAO1 exposed to P. aeruginosa PAO1 WT or PAO1*ΔhcnB* cultures in LB in the 2-petri-dish assay (Fig. S1). Each experiment was performed at least four times. Statistics correspond to two-tailed unpaired *t* test with Welch correction. N.S., not significant; ***, *P* ≤ 0.001; ****, *P* ≤ 0.0001. Download FIG S2, PDF file, 1.0 MB. Copyright © 2022 Létoffé et al. 2022 Létoffé et al. https://creativecommons.org/licenses/by/4.0/ This content is distributed under the terms of the Creative Commons Attribution 4.0 International license.

10.1128/mbio.02154-22.3 FIG S3 Complementation of the *hcnB* mutation in P. aeruginosa PAO1. (A) Graph representing the quantification of the effect of exposure of S. aureus MW2 to P. aeruginosa PAO1 WT or PAO1Δ*hcnB* mutant either carrying the empty vector pSEVA238 or p*hcnBC* in LB containing the inducer sodium benzoate (2 mM) under aerobic conditions. Bacteria were grown on 10^−5^ to 10^−8^ (grey, blue, green, and red bars, respectively) dilution spots (see Fig. S1 for setup) exposed to P. aeruginosa HCN or not. Each spot was punched out from the LB agar plate and resuspended in PBS, and the corresponding OD_600_ was determined. The fold differences observed between different conditions at comparable dilutions are indicated. They were calculated based on the ratio of the means of 4 independent quantifications at each dilution. (B) Serial dilution of S. aureus MW2 exposed to P. aeruginosa WT or PAO1*ΔhcnB* cultures in LB supplemented or not with 0.4% (wt/vol) glycine in the 2-petri-dish assay under aerobic conditions (Fig. S1). Pictures were taken after 24 h of incubation at 37°C under aerobic conditions. Each experiment was performed at least three times. (C and D) Same as panels A and B, except that the experiments were performed under microaerobic conditions. Statistics correspond to a two-tailed unpaired *t* test with Welch correction. N.S., not significant; ****, *P* ≤ 0.0001. Download FIG S3, PDF file, 2.4 MB. Copyright © 2022 Létoffé et al. 2022 Létoffé et al. https://creativecommons.org/licenses/by/4.0/ This content is distributed under the terms of the Creative Commons Attribution 4.0 International license.

### Production of HCN is widespread among Pseudomonas aeruginosa strains and active against diverse S. aureus isolates.

To determine whether HCN production is a widespread P. aeruginosa property, we first determined that a S. aureus MW2 *srrAB* mutant lacking the SrrAB global regulator of the transition from aerobic to anaerobic respiration (48) displayed a 22-fold-increased sensitivity to HCN produced by P. aeruginosa PAO1 under aerobic conditions, compared to WT MW2 (Fig. S4). We therefore used this HCN-sensitive mutant strain as an indicator strain and exposed the S. aureus *srrAB* mutant to a panel of laboratory and clinical P. aeruginosa strains, many of them isolated from airway infections (Table 1). We showed that, despite variations, all tested strains aerially inhibited S. aureus *srrAB* under aerobic conditions, even in the absence of glycine (Fig. S5C). Moreover, P. aeruginosa strains that led to a minimal reduction of S. aureus growth showed a strong growth inhibition phenotype when grown in the presence of glycine, indicative of an increase in HCN production (Fig. S5D). In addition, we also showed that the production of HCN by P. aeruginosa PAO1 in the presence of glycine inhibited the growth of a wide panel of distinct pathogenic S. aureus strains (Fig. S6), confirming HCN-mediated growth inhibition at distance over a broad range of S. aureus strains.

**TABLE 1:** Plasmids and strains used in the study

Plasmid or strain	Relevant characteristics or origin	Reference or source
Plasmids		
pMRP9-1	*gfp-*expressing plasmid	71
pSEVA238	XylS/Pm regulator/promoter expression vector inducible by sodium benzoate (2 mM)	69
phcnBC	pSEVA238 derivative plasmid expressing *hcnBC*	This study
P. aeruginosa strains		
PAO1	Wound infection, Melbourne, Australia	46
PAO1*gfp*	Green fluorescent PAO1 containing pMRP9-1	This study
PAO1Δ*hcnB*	HCN-deficient *ΔhcnB* strain	University of Washington Genome Center, gift from O. Lesouhaitier
PAO1Δ*hcnB gfp*	Green fluorescent PAO1Δ*hcnB* containing pMRP9-1	This study
PA14	Clinical isolate from burn patients	72
PAK	Virulent strain sensitive to Pf phage	73
7508	Bronchial secretion	74
8931	Lung transplant	74
9854	Nasal swab	74
11989	Tonsil swab	74
12269	Sputum	74
13305	Bronchial secretion	74
Psae1152	Drainage catheter	74
Psae1471	Respiratory tract	74
Psae1659	Respiratory tract	74
Psae1716	Blood	74
Psae1928	Respiratory tract	74
Psae2328	Urine	74
BJN8	Catheter-related bloodstream infection from Beaujon Hospital, patient 8	75
BJN33	Catheter-related bloodstream infection from Beaujon Hospital, patient 33	75
BJN53	Catheter-related bloodstream infection from Beaujon Hospital, patient 53	75
BJN66	Catheter-related bloodstream infection from Beaujon Hospital, patient 66	75
S. aureus strains		
MW2	Community acquired, methicillin resistant	76
MW2 Δ*srrAB*	Deletion of *srrAB* genes	Gift from I. Lasa (77)
15981	Biofilm-forming strain isolated at the Microbiology Department of the University Clinics of Navarra	78
COL	Initially isolated from the operating theater in a hospital in Colindale, England, in the early 1960s	79
Newman	Isolated in 1952 from a human infection	80
Xen36		Caliper Life Sciences
HG001	Highly virulent strain derivative of NCTC 8325 originally used to propagate bacteriophage 47	81
N315	Hospital-acquired methicillin-resistant isolated in 1982 from a pharyngeal smear from a patient in Japan	82
USA300LAC	Epidemic community-associated methicillin-resistant isolated from the Los Angeles County jail	83
USA300LAC*dsred*		84
V329	Biofilm-forming bovine mastitis isolate	85

10.1128/mbio.02154-22.4 FIG S4 S. aureus MW2 *srrAB* mutant displayed increased sensitivity to HCN. (Top) Effect of exposure of S. aureus MW2 or MW2*ΔsrrAB* to HCN produced by P. aeruginosa WT in LB under aerobic conditions. Bacteria were grown on 10^−6^ to 10^−8^ (blue, green, and red bars, respectively) dilution spots (see Fig. S1 for setup) exposed to P. aeruginosa HCN or not. Each spot was punched out from the LB agar plate and resuspended in PBS, and the corresponding OD_600_ was determined. The fold differences observed between different conditions at comparable dilutions were calculated based on the ratio of the means of 4 independent quantifications at each dilution. (Bottom) Serial dilution of S. aureus MW2 or MW2*ΔsrrAB* exposed to HCN produced by P. aeruginosa WT in LB under aerobic conditions in the 2-petri-dish assay (Fig. S1). Pictures were taken after 24 h of incubation at 37°C under aerobic conditions. Statistics correspond to a two-tailed unpaired *t* test with Welch correction. Each experiment was performed at least four times. ****, *P* ≤ 0.0001. Download FIG S4, PDF file, 0.8 MB. Copyright © 2022 Létoffé et al. 2022 Létoffé et al. https://creativecommons.org/licenses/by/4.0/ This content is distributed under the terms of the Creative Commons Attribution 4.0 International license.

10.1128/mbio.02154-22.5 FIG S5 Production of HCN is widespread among P. aeruginosa strains and clinical isolates. (A) Growth of serial dilutions of the S. aureus MW2 *srrAB* mutant upon exposure to LB (A) or to a panel of laboratory (B) and clinical (C) P. aeruginosa strains grown in LB after 24 h incubation at 37°C under aerobic conditions, using the 2-petri-dish assay described in Fig. S1. (D) For strains that showed only partially reduced S. aureus growth, 0.4% (wt/vol) glycine was also added in LB medium. Each experiment was performed at least three times. The dilutions are indicated only on the visible growth spots. Download FIG S5, PDF file, 2.2 MB. Copyright © 2022 Létoffé et al. 2022 Létoffé et al. https://creativecommons.org/licenses/by/4.0/ This content is distributed under the terms of the Creative Commons Attribution 4.0 International license.

10.1128/mbio.02154-22.6 FIG S6 Growth inhibition of a panel of S. aureus strains upon aerial exposure to P. aeruginosa PAO1 culture. The growth of serial dilution of a panel of S. aureus strains upon exposure to P. aeruginosa PAO1 cultures in LB supplemented with 0.4% (wt/vol) glycine after 24 h incubation at 37°C under aerobic conditions, using the 2-petri-dish assay as described in Fig. S1. Each experiment was performed at least three times. Download FIG S6, PDF file, 2.9 MB. Copyright © 2022 Létoffé et al. 2022 Létoffé et al. https://creativecommons.org/licenses/by/4.0/ This content is distributed under the terms of the Creative Commons Attribution 4.0 International license.

### Microaerobiosis enhances P. aeruginosa HCN production.

P. aeruginosa HCN production is regulated by quorum sensing (36). Consistently, we observed an increase of the HCN signal during the transition from exponential to stationary phase, at culture densities equivalent to an optical density at 600 nm (OD_600_) of >2 (Fig. 1C). We also confirmed that the P. aeruginosa HCN production was enhanced under microaerobic conditions (0.4 to 0.8% O_2_) compared to aerobic conditions (Fig. 1A and B) (33). Consistently, under these microaerobic conditions, the growth of S. aureus MW2 exposed to P. aeruginosa PAO1 was reduced by 4-fold, compared to a 1.4-fold reduction upon exposure in aerobic conditions (Fig. 2B and D). These results show that microaerobic conditions led to higher HCN production in P. aeruginosa PAO1, which correlated with a strong growth reduction of S. aureus MW2 under these conditions.

### Production of HCN impairs S. aureus growth in *in vitro* mixed biofilms.

**FIG 3:**
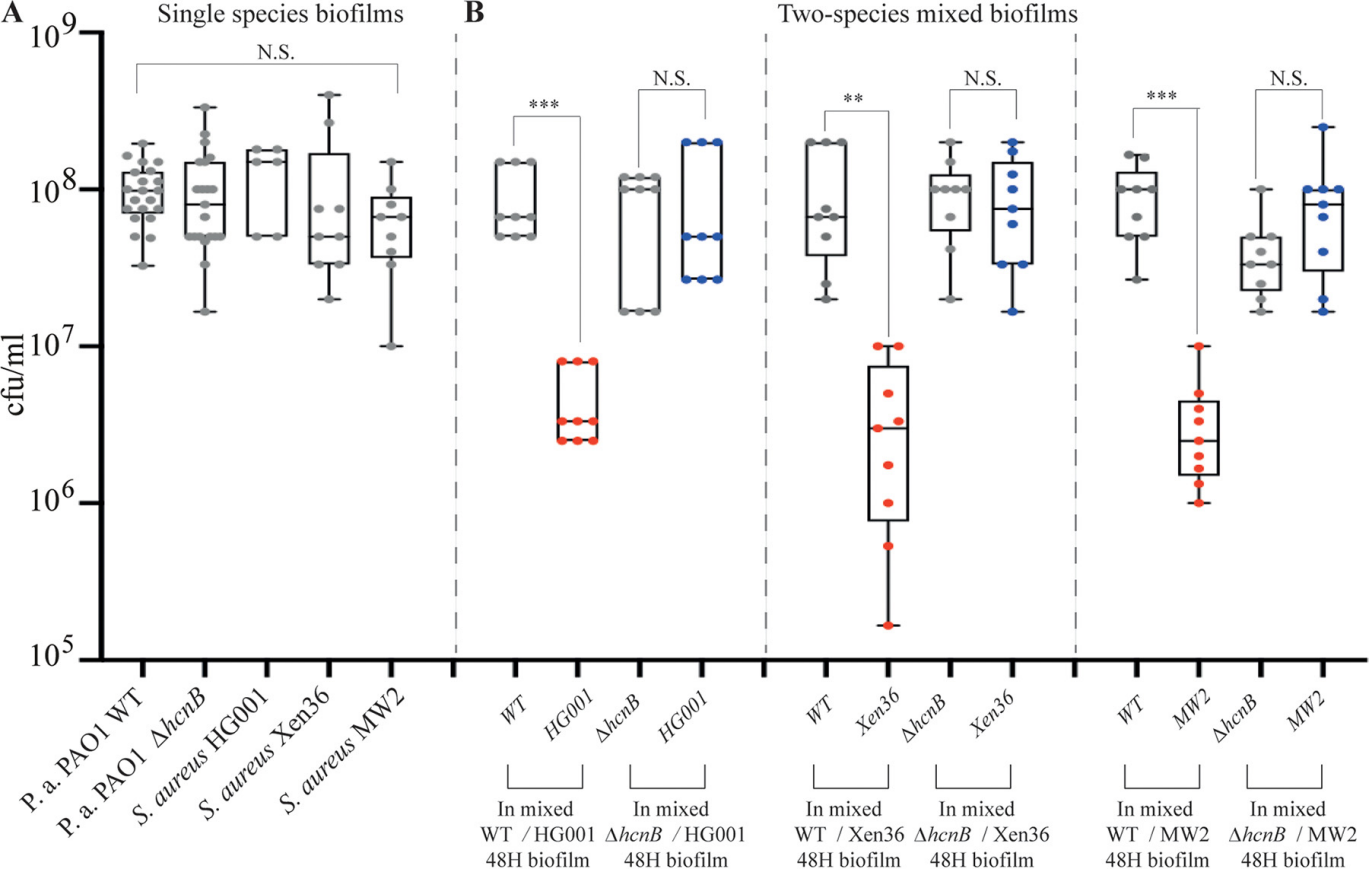
Production of HCN controls S. aureus growth in *in vitro* mixed biofilms. (A) Number of CFU in single-species biofilms grown in LB medium in continuous-flow microfermentors for 48 h at 37°C. (B) Number of CFU in two-species mixed biofilms. Each S. aureus strain was mixed with either WT P. aeruginosa PAO1 or its *ΔhcnB* mutant at a 1:1 ratio. The biofilms were grown in LB medium in continuous-flow microfermentors for 48 h at 37°C. Statistics correspond to a two-tailed unpaired *t* test with Welch correction. N.S., not significant; **, *P* ≤ 0.01; ***, *P* ≤ 0.001.

Our results suggested that HCN production could contribute to the dynamics of P. aeruginosa-S. aureus competition. Considering that microaerobic conditions prevail within multispecies biofilms (49), we hypothesized that P. aeruginosa HCN production in biofilms could impact S. aureus growth dynamics in mixed P. aeruginosa-S. aureus biofilms. To test this *in vitro*, we coinoculated continuous-flow biofilm microfermentors with P. aeruginosa PAO1 WT (HCN^+^) and a *ΔhcnB* (HCN^−^) mutant at a 1:1 ratio with three different S. aureus strains, including HG001, Xen36, and MW2. While all strains displayed similar individual biofilm-forming capacities (Fig. 3A), the P. aeruginosa and S. aureus proportions in the resulting two-species biofilms formed after 48 h showed that all tested S. aureus strains formed less biofilm biomass, as measured by CFU count, when mixed with WT P. aeruginosa than with the HCN-deficient mutant (Fig. 3B). Taken together, these results indicate that the production of P. aeruginosa HCN impairs S. aureus growth in mixed biofilms.

### Production of P. aeruginosa HCN controls S. aureus growth under CF-relevant conditions.

**FIG 4:**
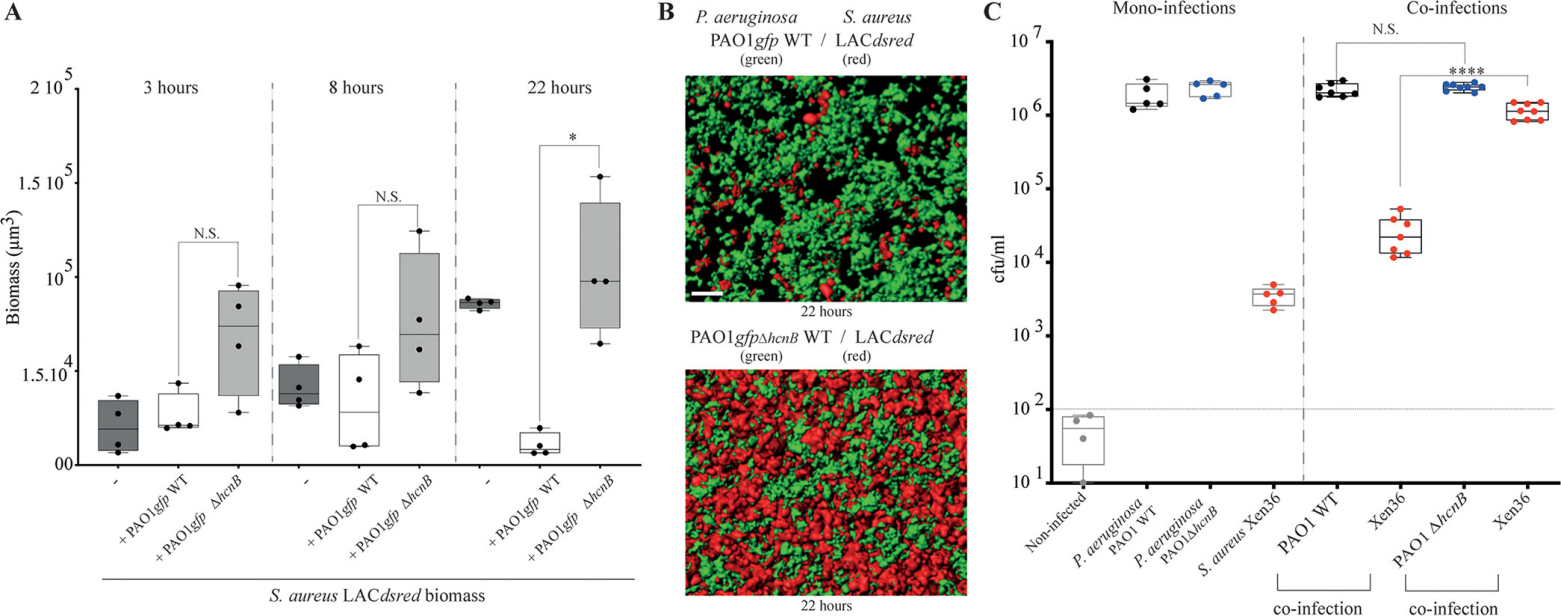
P. aeruginosa production of HCN controls S. aureus growth in CF-relevant conditions. (A) Total biomass of S. aureus aggregates as monoculture or in coinfection with the PAO1 wild type and/or the *hcnB* mutant. Isolates were cultured in SCFM2 and imaged using confocal microscopy at 3, 8, and 22 h. Statistics correspond to a two-tailed unpaired *t* test with Welch correction. N.S., not significant; *, *P* ≤ 0.05. (B) Representative rendered confocal micrograph of S. aureus and P. aeruginosa coinfection in SCFM2. (Top) Wild-type P. aeruginosa in green and S. aureus in red. (Bottom) P. aeruginosa Δ*hcnB* mutant in green and S. aureus in red. (C) *In vivo* competition experiments in mouse lungs. Monoinoculation and mixed (1:2) coinoculations of S. aureus Xen36, P. aeruginosa PAO1 WT, or the *ΔhcnB* mutant were used to infect animals. CFU were counted in the lung homogenates of mice 24 h after infection. Noninfected SOPF mice showed minimal lung bacterial contamination, with <100 CFU (horizontal dotted line). Statistics correspond to a two-tailed unpaired *t* test with Welch correction. N.S., not significant; ****, *P* ≤ 0.0001.

To test whether production of HCN could advantage P. aeruginosa over S. aureus under CF-relevant conditions, we first used synthetic CF sputum (SCFM2) medium, designed to recapitulate human CF environments (50). We first verified that P. aeruginosa HCN production (Fig. 1B and C) and S. aureus MW2 sensitivity profiles observed using LB medium were also observed with the SCFM2 medium under both aerobic and microaerobic conditions (Fig. S7). We then inoculated this medium with red fluorescent S. aureus LAC*dsrfp* alone or mixed in a 1:1 ratio with P. aeruginosa PAO1*gfp* WT (HCN^+^) or its Δ*hcnB* (HCN^−^) mutant. The comparison of the respective growth of S. aureus and P. aeruginosa revealed a strong reduction of S. aureus biomass development at 22 h (Fig. 4A) that correlated with enhanced aggregate abundance of S. aureus when coinoculated with Δ*hcnB* P. aeruginosa compared to with WT P. aeruginosa (Fig. 4B).

10.1128/mbio.02154-22.7 FIG S7 P. aeruginosa production of HCN in SCFM medium also leads to airborne inhibition of S. aureus growth. (A) Effect of exposure to P. aeruginosa HCN on S. aureus MW2 growth under SCFM aerobic conditions. Bacteria were grown on 10^−5^ to 10^−8^ (grey, blue, green, and red bars, respectively) dilution spots (see Fig. S1 for setup) and exposed to P. aeruginosa HCN or not. Each spot was punched out from the LB agar plate and resuspended in PBS, and the corresponding OD_600_ was determined. The fold differences observed between different conditions at comparable dilutions were calculated based on the ratio of the mean of 4 independent quantifications at each dilution. (B) Serial dilution of S. aureus MW2 exposed to P. aeruginosa WT or PAO1*ΔhcnB* cultures in SCFM supplemented or not with 0.4% (wt/vol) glycine in the 2-petri-dish assay (Fig. S1). No inhibition of S. aureus MW2 growth was observed when the middle small petri dish containing P. aeruginosa culture was sealed with Parafilm. Pictures were taken after 24 h of incubation at 37°C under aerobic conditions. Each experiment was performed at least three times. (C and D) Same as panels A and B, except that the experiments were performed under microaerobic conditions. Statistics correspond to a two-tailed unpaired *t* test with Welch correction. ***, *P* ≤ 0.001; ****, *P* ≤ 0.0001. Download FIG S7, PDF file, 1.8 MB. Copyright © 2022 Létoffé et al. 2022 Létoffé et al. https://creativecommons.org/licenses/by/4.0/ This content is distributed under the terms of the Creative Commons Attribution 4.0 International license.

To further test the *in vivo* impact of HCN production on S. aureus-P. aeruginosa mixed-community dynamics in the microaerobic lung airways, we performed an *in vivo* competition in mice in which lungs were intratracheally coinoculated with S. aureus Xen36 and either P. aeruginosa PAO1 WT or PAO1Δ*hcnB*. Mouse lungs infected individually showed that P. aeruginosa colonizes this environment better than S. aureus Xen36 (Fig. 4C, left). The comparison of the number of CFU extracted from mouse lungs coinoculated in a 2:1 ratio (P. aeruginosa-S. aureus) showed that whereas the presence of WT P. aeruginosa stimulates S. aureus growth, a further 2-log increase of S. aureus CFU was observed in the presence of the PAO1 *ΔhcnB* mutant (HCN^−^) (Fig. 4C). Taken together, our results demonstrate that HCN production by P. aeruginosa controls S. aureus colonization in coinfected mouse lungs and other microaerobic biofilm-like environments.

## DISCUSSION

In this study, we showed that airborne HCN produced by a wide range of P. aeruginosa clinical strains is enhanced under microaerobic conditions and inhibits various S. aureus isolates *in vitro*, controlling S. aureus colonization in polymicrobial biofilms. This occurs both in a CF sputum medium and in an *in vivo* mouse model of pulmonary coinfection. A number of bacterial infections are characterized by the development of polymicrobial communities in which complex interactions between bacteria can influence the outcome of diseases (12, 17, 43, 51). Colonization of the lungs during CF is one of the best examples of polymicrobial infection that is characterized by excessive mucus production in airways and decreased mucosal clearance (52). This favors lung colonization by bacterial pathogens, including P. aeruginosa, S. aureus, nontypeable Haemophilus influenzae, and Burkholderia cepacia, where prevalence varies with the age and treatments of CF patients (52–55). These airway infections are difficult to eradicate despite aggressive antibiotic therapy (56, 57) and are associated with inflammation, leading to a progressive decline in lung functions and, ultimately, to respiratory failure (53, 57–59).

Interactions between microorganisms have been shown to be key determinants of their distribution and activity in most ecosystems (12, 58), with several P. aeruginosa secreted molecules having been shown to inhibit S. aureus’s growth (16, 19, 25, 26, 60). In contrast to local competition driven by short-range diffusion (<10 μm) of most inhibitory metabolic products, volatile HCN produced by Pseudomonas and a number of other bacteria could play an important role in the spatial organization of microbial communities (31). HCN could indeed contribute to both local and distant airborne competition between microorganisms in physically heterogeneous solid, liquid, and gaseous environments, such as the lungs and other organic tissues.

Our results are of pathophysiological relevance, since CF lungs are known to present microaerobic areas and to be commonly associated with the presence of S. aureus and P. aeruginosa multispecies biofilms, reaching high P. aeruginosa cell density, two conditions that have been shown to induce *hcnABC* gene expression and subsequent HCN production (30, 31, 34, 36, 61). HCN was indeed previously detected in the sputum and bronchoalveolar lavage fluids of CF patients infected by P. aeruginosa, and the measure of HCN levels in lungs of CF patients has been used as a noninvasive breath test to diagnose P. aeruginosa infection (44, 62–64). This suggests that the levels of HCN produced by P. aeruginosa in the lung environment could be sufficient to poison aerobic metabolism and growth, excluding competitors from P. aeruginosa ecological niches (39, 44).

S. aureus had been regarded as one of the initial microbial colonizers of CF patients’ airways before being displaced by P. aeruginosa (14, 20), and our results support the hypothesis that metabolic poisoning upon HCN production could be a key determinant of Pseudomonas distribution in the lung upon exclusion of S. aureus in mixed *in vitro and in vivo* polymicrobial biofilms (19, 28, 34, 39). However, P. aeruginosa PAO1 was also shown to reduce its toxicity toward S. aureus or to facilitate S. aureus microcolony formation through alginate production, thus promoting the coexistence of these two bacteria (13, 65–67). Consistently, we observed that although production of HCN by PAO1 WT controlled the number of S. aureus organisms recovered from coinoculated lungs compared to coinoculation with PAO1*ΔhcnB*, there was a 1,000-fold increase in S. aureus abundance when monoinoculation and coinoculation with PAO1Δ*hcnB* were compared (Fig. 4C). This indicates that, in the absence of HCN, S. aureus growth is stimulated by P. aeruginosa, which further emphasizes that these two bacteria could engage in complex negative and positive interactions *in vivo* (67, 68).

Our study therefore contributes to a better understanding of molecular actors in the P. aeruginosa-S. aureus competition in biofilms, sputum medium (SCFM2), and *in vivo* lung colonization, all three environments being relevant in the context of CF airway infection. Whereas further studies will be required to tease out the respective ecological contribution of HCN and other P. aeruginosa factors to competition with S. aureus, our results further illustrate the remarkable ability of P. aeruginosa to adapt and thrive in multispecies communities. The identification of a volatile compound-based mechanism potentially underlying the dynamic shift from S. aureus to P. aeruginosa dominance in polymicrobial infection opens new perspectives for the management or monitoring of P. aeruginosa infections in lower-lung airway infections and other polymicrobial disease contexts.

## MATERIALS AND METHODS

### Bacterial strains, plasmids, and growth conditions.

Bacterial strains and plasmids used in this study are listed in Table 1. All experiments were performed in lysogeny broth (LB) or SCFM2 (50) medium, supplemented or not with 0.4% (wt/vol) glycine and incubated at 37°C. All chemicals were purchased from Sigma-Aldrich.

### Plasmid expressing *hcnBC* for complementation experiments.

To build a plasmid expressing *hcnBC*, we amplified *hcnBC* by PCR and cloned these two genes into pSEVA238 (69) by the isothermal Gibson Assembly method (New England Biolabs, Ipswich, MA, USA) using the primers hcnBC-pSEVA-FOR (GCGCGAATTCGAGCTCGGTACCCGGatgaacctgcgcccggtg), hcnBC-pSEVA-REV (CTGCAGGTCGACTCTAGAGGATCCctagcaggccgcgaccgccacg), pSEVA vect REV (CCGGGTACCGAGCTCGAATTCGCGC), pSEVA vect FOR (GGATCCTCTAGAGTCGACCTGCAG), Kana FOR (GAGCCATATTCAGCGTGAAACGAG), and Kana REV (CATCCAGCATCAGATGAAATTGC). The plasmid obtained, p*hcnBC*, was verified by PCR with specific primers and DNA sequencing.

In p*hcnBC*, *hcnBC* are placed under the control of the XylS/Pm regulator/promoter system, which is inducible by sodium benzoate (2 mM). p*hcnBC* and the empty pSEVA238 vector were introduced into PAO1 or the *hcnB* mutant by electroporation. For this, a 10-mL overnight culture of P. aeruginosa in LB medium was centrifuged at 4,000 × *g* for 5 min at room temperature. The cell pellet was washed and resuspended in 1 mL of 300 mM sucrose and transferred to a 2-mL reaction tube, followed by centrifugation at 15,000 × *g* for 1 min. The supernatant was carefully discarded, and the washing steps were repeated twice. In the final step, the cell pellet was resuspended in 0.4 mL of 300 mM sucrose. An aliquot of 0.1 mL of the resulting suspension was mixed with 200 ng of each plasmid and transferred to a 0.1-cm-gap electroporation cuvette. After application of a pulse (settings: 25 μF, 200 Ω, and 2.5 kV on a Bio-Rad GenePulser XCell), 1 mL of super optimal broth (SOC) medium was added, followed by a 2 h of incubation at 37°C with rotational agitation. The biomass was then concentrated to 0.1 mL by centrifugation, plated onto LB agar plates supplemented with 400 μg/mL kanamycin, and incubated at 37°C. Controls included cells pulsed without added DNA.

### Screening for volatile-mediated HCN phenotypes.

To evaluate the activity of HCN released by bacterial liquid culture on recipient test bacteria, a lidless 3.5-cm petri dish was placed inside a 9-cm petri dish, and the external ring was filled with 20 mL of 1.5% LB agar (Fig. S1A) (4). Tested recipient bacteria were spotted as 20-μL drops of 10^−4^ to 10^−8^ serial dilutions of an overnight culture adjusted to an OD_600_ of 1, and P. aeruginosa bacterial liquid cultures releasing volatile HCN or not were adjusted to an OD_600_ of 3 and introduced into the middle of an uncovered petri dish. The large petri dish was then closed and incubated for 24 h at 37°C, under aerobic or microaerobic conditions. Exposure to microaerobic conditions (0.4 to 0.8% O_2_) was performed in a C400M Ruskinn anaerobic-microaerobic station.

### Detection of HCN production.

Semiquantitative determination of the levels of HCN production used a method adapted from previous studies (47). Briefly, using the setup described in Fig. S1A, Whatman chromatography paper soaked in HCN detection reagent containing copper(II) ethyl acetoacetate (100 mg) and 4,4′-methylenebis-(*N*,*N*-dimethylaniline) (100 mg) solubilized in 20 mL chloroform was laid for the duration of the incubation on the surface of the central, uncovered 3.5-cm petri dish containing bacterial liquid culture releasing volatile HCN or not. The large petri dish was then closed and incubated for 24 h at 37°C in aerobic or microaerobic conditions. Exposure under microaerobic conditions (0.4 to 0.8% O_2_) was performed in a C400M Ruskinn anaerobic-microaerobic station. The level of HCN was evaluated based on the intensity of blue color resulting from exposure to bacterial HCN.

### Colony quantification.

We quantified colonies growing on serial dilutions spotted on plates, in quadruplicate, as indicated in Fig. S1. After 24 h growth and exposure, each spot was reproducibly punched out from the agar plate, transferred into a tube containing 3 mL of phosphate-buffered saline (PBS), and vortexed for 1 min. One milliliter of the suspension was then transferred to a microcuvette, and the corresponding OD_600_ was determined using a Bio-Rad SmartSpec 3000 spectrophotometer. Each quantification was performed 4 times independently.

### Test in an SCFM2 artificial sputum model.

Green fluorescent P. aeruginosa (WT or HCN mutant) carrying pMRP9-1 and S. aureus expressing DsRed red fluorescent protein (Table 1) were grown overnight in tryptic soy broth (TSB). Cells were washed twice and resuspended in PBS. The OD_600_ was measured with a spectrophotometer, and washed bacterial cultures were inoculated into SCFM2 at an OD_600_ of 0.05 (~10^7^ CFU/mL) individually or in combination. Cultures were vortexed for 5 to 10 s to disperse bacterial cells in SCFM2. Five hundred microliters of inoculated SCFM2 were then transferred into each well of four-well microchamber slides (Lab-Tek; Nunc) and incubated under static conditions at 37°C.

### Biofilm competition experiments in microfermentors.

Continuous-flow biofilm microfermentors containing a removable glass spatula were used as described in reference 70 (see also https://research.pasteur.fr/en/tool/biofilm-microfermenters/). Medium flow was adjusted to 60 mL/h with internal bubbling agitation with filter-sterilized compressed air to minimize planktonic growth over biofilm development. Inoculation was performed by removing the glass spatula from the microfermentor and by dipping it for 10 min in overnight LB cultures of S. aureus and P. aeruginosa strains mixed at a 1:1 ratio (OD_600_ = 1). The spatula was then reintroduced into the microfermentor; the resulting 48-h mixed biofilms grown on the fermentor spatula were recovered, and corresponding serial dilutions were plated on LB agar (all bacteria) and/or on P. aeruginosa-selective pseudomonas isolation agar (PIA) plates and S. aureus-selective mannitol salt agar (MSA) plates.

**(i) Imaging.** All images were acquired with Zeiss LSM 700 and LSM 880 confocal laser scanning microscopes utilizing Zen image capture software. Bacterial cells were visualized via green fluorescent protein (GFP) with an excitation wavelength of 488 nm and an emission wavelength of 509 nm, via DsRed with an excitation wavelength of 587 nm and an emission wavelength of 610 nm, or with a 63× oil immersion objective. SCFM2 images were acquired by producing 512- by 512-pixel (0.26- by 0.26-μm pixels) 8-bit z-stack images that were 100 μm from the base of the coverslip. The total volumes of 100-μm z-stack images were 1,822.5 mm^3^. Control images of uninoculated SCFM2 were acquired by using identical settings to determine the background fluorescence for image analysis.

**(ii) Image analysis.** All imaging was performed with identical image capture settings. To determine the background fluorescence in SCFM2, a histogram of detected DsRed and GFP fluorescence was produced in Imaris v 8.3.1 (Bitplane) for uninoculated SCFM2, and the average of the three highest voxel values was determined as the background fluorescence. Averaging across all of the control images, this value was then subtracted from all experimental images with Imaris. For aggregate and biomass quantification in SCFM2, isosurfaces were produced for all remaining voxels after background subtraction with the “surpass” module in Imaris. To detect individual aggregates, the “split objects” option in Imaris was enabled. Objects that were ≥0.5 and ≤5.0 μm^3^ were categorized as dispersed biomass, and objects that were >5.0 μm^3^ were categorized as aggregated biomass. Detected aggregate isosurfaces were then ordered by volume. The total biomass (all voxels detected) was calculated by the sum of all individual aggregate object volumes plus those of dispersed objects. Using the “vantage” module in Imaris, average aggregate volume and number of aggregates were calculated (data not shown). For each experiment, the tested variable and control were imaged at 3 different coordinates chosen at random, providing three technical replicates per well visualized. All image data were exported into Microsoft Excel 2016, and graphs were generated with GraphPad Prism 7.

### *In vivo* experiments.

Specific opportunistic-pathogen-free (SOPF) BALB/c mice (male, 7 weeks, in particular free of detectable S. aureus and P. aeruginosa strains) were ordered from Janvier Labs (France) and housed in the Institut Pasteur animal facilities. All experiments were approved by the Ethics Committee of Institut Pasteur (reference 2014-0014). Mice were infected intratracheally as described previously (19). In brief, mice were anesthetized by intraperitoneal injection of ketamine (Imalgene 1000; 90 mg/kg)-xylazine (Rompun; 10 mg/kg) suspended in PBS. The anesthetized animals (5 mice per group for monoinfection and 7 mice per group for coinfection) were subjected to noninvasive intratracheal catheterization through which P. aeruginosa PAO1 or PAO1Δ*hncB* (1 × 10^6^ CFU) and/or S. aureus Xen36 (5 × 10^5^ CFU) suspended in 50 μL of PBS was introduced to initiate the infection. Xen36 is derived from the parental strain S. aureus ATCC 49525 (Wright), which was isolated from a bacteremic patient and specifically developed for *in vivo* rodent infection models. For coinfection, a P. aeruginosa*/*S. aureus ratio of 2:1 was optimized to allow detection in lung homogenates and to avoid mouse mortality within 24 h. Twenty-four hours postinfection, the animals were sacrificed by intraperitoneal injection of a lethal dose of pentobarbital. The lungs were harvested and homogenized as described previously (19). The lung homogenates were serially diluted, and the number of bacterial CFU in the lung was determined by plating and counting bacteria on LB agar (all bacteria) and/or on P. aeruginosa-selective PIA plates and S. aureus-selective MSA plates.

### Statistical analysis.

The two-tailed unpaired *t* test with Welch correction analyses was performed using Prism 9.0 for Mac OS X (GraphPad Software). Each experiment was performed at least three times.

## References

[1] Schulz-Bohm K, Martín-Sánchez L, Garbeva P. 2017. Microbial volatiles: small molecules with an important role in intra- and inter-kingdom interactions. Front Microbiol 8:2484. doi:10.3389/fmicb.2017.02484. 29312193 PMC5733050

[2] Weisskopf L, Schulz S, Garbeva P. 2021. Microbial volatile organic compounds in intra-kingdom and inter-kingdom interactions. Nat Rev Microbiol 19:391–404. doi:10.1038/s41579-020-00508-1. 33526910

[3] Kai M, Haustein M, Molina F, Petri A, Scholz B, Piechulla B. 2009. Bacterial volatiles and their action potential. Appl Microbiol Biotechnol 81:1001–1012. doi:10.1007/s00253-008-1760-3. 19020812

[4] Bernier SP, Letoffe S, Delepierre M, Ghigo JM. 2011. Biogenic ammonia modifies antibiotic resistance at a distance in physically separated bacteria. Mol Microbiol 81:705–716. doi:10.1111/j.1365-2958.2011.07724.x. 21651627

[5] Kesarwani M, Hazan R, He J, Que Y-A, Que Y, Apidianakis Y, Lesic B, Xiao G, Dekimpe V, Milot S, Deziel E, Lépine F, Rahme LG. 2011. A quorum sensing regulated small volatile molecule reduces acute virulence and promotes chronic infection phenotypes. PLoS Pathog 7:e1002192. doi:10.1371/journal.ppat.1002192. 21829370 PMC3150319

[6] Kim KS, Lee S, Ryu CM. 2013. Interspecific bacterial sensing through airborne signals modulates locomotion and drug resistance. Nat Commun 4:1809. doi:10.1038/ncomms2789. 23651997

[7] Bos LDJ, Sterk PJ, Schultz MJ. 2013. Volatile metabolites of pathogens: a systematic review. PLoS Pathog 9:e1003311. doi:10.1371/journal.ppat.1003311. 23675295 PMC3649982

[8] Audrain B, Farag MA, Ryu CM, Ghigo JM. 2015. Role of bacterial volatile compounds in bacterial biology. FEMS Microbiol Rev 39:222–233. doi:10.1093/femsre/fuu013. 25725014

[9] Govan JR, Deretic V. 1996. Microbial pathogenesis in cystic fibrosis: mucoid Pseudomonas aeruginosa and Burkholderia cepacia. Microbiol Rev 60:539–574. doi:10.1128/mr.60.3.539-574.1996. 8840786 PMC239456

[10] Harrison F. 2007. Microbial ecology of the cystic fibrosis lung. Microbiology (Reading) 153:917–923. doi:10.1099/mic.0.2006/004077-0. 17379702

[11] Hogan DA, Willger SD, Dolben EL, Hampton TH, Stanton BA, Morrison HG, Sogin ML, Czum J, Ashare A. 2016. Analysis of lung microbiota in bronchoalveolar lavage, protected brush and sputum samples from subjects with mild-to-moderate cystic fibrosis lung disease. PLoS One 11:e0149998. doi:10.1371/journal.pone.0149998. 26943329 PMC4778801

[12] Stacy A, McNally L, Darch SE, Brown SP, Whiteley M. 2016. The biogeography of polymicrobial infection. Nat Rev Microbiol 14:93–105. doi:10.1038/nrmicro.2015.8. 26714431 PMC5116812

[13] Limoli DH, Whitfield GB, Kitao T, Ivey ML, Davis MR, Grahl N, Hogan DA, Rahme LG, Howell PL, O’Toole GA, Goldberg JB. 2017. Pseudomonas aeruginosa alginate overproduction promotes coexistence with Staphylococcus aureus in a model of cystic fibrosis respiratory infection. mBio 8:e00186-17. doi:10.1128/mBio.00186-17. 28325763 PMC5362032

[14] Woods PW, Haynes ZM, Mina EG, Marques CNH. 2018. Maintenance of S. aureus in co-culture with P aeruginosa while growing as biofilms. Front Microbiol 9:3291. doi:10.3389/fmicb.2018.03291. 30687276 PMC6333908

[15] Niggli S, Kümmerli R. 2020. Strain background, species frequency, and environmental conditions are important in determining Pseudomonas aeruginosa and Staphylococcus aureus population dynamics and species coexistence. Appl Environ Microbiol 86:e00962-20. doi:10.1128/AEM.00962-20. 32651205 PMC7480381

[16] Hoffman LR, Déziel E, D’Argenio DA, Lépine F, Emerson J, McNamara S, Gibson RL, Ramsey BW, Miller SI. 2006. Selection for Staphylococcus aureus small-colony variants due to growth in the presence of Pseudomonas aeruginosa. Proc Natl Acad Sci USA 103:19890–19895. doi:10.1073/pnas.0606756104. 17172450 PMC1750898

[17] Hibbing ME, Fuqua C, Parsek MR, Peterson SB. 2010. Bacterial competition: surviving and thriving in the microbial jungle. Nat Rev Microbiol 8:15–25. doi:10.1038/nrmicro2259. 19946288 PMC2879262

[18] Baldan R, Cigana C, Testa F, Bianconi I, De Simone M, Pellin D, Di Serio C, Bragonzi A, Cirillo DM. 2014. Adaptation of Pseudomonas aeruginosa in cystic fibrosis airways influences virulence of Staphylococcus aureus in vitro and murine models of co-infection. PLoS One 9:e89614. doi:10.1371/journal.pone.0089614. 24603807 PMC3945726

[19] Pernet E, Guillemot L, Burgel PR, Martin C, Lambeau G, Sermet-Gaudelus I, Sands D, Leduc D, Morand PC, Jeammet L, Chignard M, Wu Y, Touqui L. 2014. Pseudomonas aeruginosa eradicates Staphylococcus aureus by manipulating the host immunity. Nat Commun 5:5105. doi:10.1038/ncomms6105. 25290234

[20] Hotterbeekx A, Kumar-Singh S, Goossens H, Malhotra-Kumar S. 2017. In vivo and In vitro interactions between Pseudomonas aeruginosa and Staphylococcus spp. Front Cell Infect Microbiol 7:106. doi:10.3389/fcimb.2017.00106. 28421166 PMC5376567

[21] Machan ZA, Taylor GW, Pitt TL, Cole PJ, Wilson R. 1992. 2-Heptyl-4-hydroxyquinoline N-oxide, an antistaphylococcal agent produced by Pseudomonas aeruginosa. J Antimicrob Chemother 30:615–623. doi:10.1093/jac/30.5.615. 1493979

[22] Kessler E, Safrin M, Olson JC, Ohman DE. 1993. Secreted LasA of Pseudomonas aeruginosa is a staphylolytic protease. J Biol Chem 268:7503–7508. doi:10.1016/S0021-9258(18)53203-8. 8463280

[23] Brint JM, Ohman DE. 1995. Synthesis of multiple exoproducts in Pseudomonas aeruginosa is under the control of RhlR-RhlI, another set of regulators in strain PAO1 with homology to the autoinducer-responsive LuxR-LuxI family. J Bacteriol 177:7155–7163. doi:10.1128/jb.177.24.7155-7163.1995. 8522523 PMC177595

[24] Bharali P, Saikia JP, Ray A, Konwar BK. 2013. Rhamnolipid (RL) from Pseudomonas aeruginosa OBP1: a novel chemotaxis and antibacterial agent. Colloids Surf B Biointerfaces 103:502–509. doi:10.1016/j.colsurfb.2012.10.064. 23261573

[25] Nguyen AT, Oglesby-Sherrouse AG. 2015. Spoils of war: iron at the crux of clinical and ecological fitness of Pseudomonas aeruginosa. Biometals 28:433–443. doi:10.1007/s10534-015-9848-6. 25790779

[26] Biswas L, Götz F. 2021. Molecular mechanisms of Staphylococcus and Pseudomonas interactions in cystic fibrosis. Front Cell Infect Microbiol 11:824042. doi:10.3389/fcimb.2021.824042. 35071057 PMC8770549

[27] Goeminne PC, Vandendriessche T, Van Eldere J, Nicolai BM, Hertog ML, Dupont LJ. 2012. Detection of Pseudomonas aeruginosa in sputum headspace through volatile organic compound analysis. Respir Res 13:87. doi:10.1186/1465-9921-13-87. 23031195 PMC3489698

[28] Zdor RE. 2015. Bacterial cyanogenesis: impact on biotic interactions. J Appl Microbiol 118:267–274. doi:10.1111/jam.12697. 25410133

[29] Elmassry MM, Piechulla B. 2020. Volatilomes of bacterial infections in humans. Front Neurosci 14:257. doi:10.3389/fnins.2020.00257. 32269511 PMC7111428

[30] Castric PA. 1975. Hydrogen cyanide, a secondary metabolite of Pseudomonas aeruginosa. Can J Microbiol 21:613–618. doi:10.1139/m75-088. 164997

[31] Blumer C, Haas D. 2000. Mechanism, regulation, and ecological role of bacterial cyanide biosynthesis. Arch Microbiol 173:170–177. doi:10.1007/s002039900127. 10763748

[32] Knowles CJ, Bunch AW. 1986. Microbial cyanide metabolism. Adv Microb Physiol 27:73–111. doi:10.1016/s0065-2911(08)60304-5. 3532718

[33] Laville J, Blumer C, Von Schroetter C, Gaia V, Défago G, Keel C, Haas D. 1998. Characterization of the hcnABC gene cluster encoding hydrogen cyanide synthase and anaerobic regulation by ANR in the strictly aerobic biocontrol agent Pseudomonas fluorescens CHA0. J Bacteriol 180:3187–3196. doi:10.1128/JB.180.12.3187-3196.1998. 9620970 PMC107821

[34] Williams HD, Zlosnik JE, Ryall B. 2007. Oxygen, cyanide and energy generation in the cystic fibrosis pathogen Pseudomonas aeruginosa. Adv Microb Physiol 52:1–71. doi:10.1016/S0065-2911(06)52001-6. 17027370

[35] Blier AS, Vieillard J, Gerault E, Dagorn A, Varacavoudin T, Le Derf F, Orange N, Feuilloley M, Lesouhaitier O. 2012. Quantification of Pseudomonas aeruginosa hydrogen cyanide production by a polarographic approach. J Microbiol Methods 90:20–24. doi:10.1016/j.mimet.2012.04.005. 22537820

[36] Pessi G, Haas D. 2000. Transcriptional control of the hydrogen cyanide biosynthetic genes hcnABC by the anaerobic regulator ANR and the quorum-sensing regulators LasR and RhlR in Pseudomonas aeruginosa. J Bacteriol 182:6940–6949. doi:10.1128/JB.182.24.6940-6949.2000. 11092854 PMC94819

[37] Carterson AJ, Morici LA, Jackson DW, Frisk A, Lizewski SE, Jupiter R, Simpson K, Kunz DA, Davis SH, Schurr JR, Hassett DJ, Schurr MJ. 2004. The transcriptional regulator AlgR controls cyanide production in Pseudomonas aeruginosa. J Bacteriol 186:6837–6844. doi:10.1128/JB.186.20.6837-6844.2004. 15466037 PMC522194

[38] Cody WL, Pritchett CL, Jones AK, Carterson AJ, Jackson D, Frisk A, Wolfgang MC, Schurr MJ. 2009. Pseudomonas aeruginosa AlgR controls cyanide production in an AlgZ-dependent manner. J Bacteriol 191:2993–3002. doi:10.1128/JB.01156-08. 19270096 PMC2681793

[39] Anderson RD, Roddam LF, Bettiol S, Sanderson K, Reid DW. 2010. Biosignificance of bacterial cyanogenesis in the CF lung. J Cyst Fibros 9:158–164. doi:10.1016/j.jcf.2009.12.003. 20156704

[40] Gallagher LA, Manoil C. 2001. Pseudomonas aeruginosa PAO1 kills Caenorhabditis elegans by cyanide poisoning. J Bacteriol 183:6207–6214. doi:10.1128/JB.183.21.6207-6214.2001. 11591663 PMC100099

[41] Broderick KE, Chan A, Balasubramanian M, Feala J, Reed SL, Panda M, Sharma VS, Pilz RB, Bigby TD, Boss GR. 2008. Cyanide produced by human isolates of Pseudomonas aeruginosa contributes to lethality in Drosophila melanogaster. J Infect Dis 197:457–464. doi:10.1086/525282. 18199034

[42] Blom D, Fabbri C, Eberl L, Weisskopf L. 2011. Volatile-mediated killing of Arabidopsis thaliana by bacteria is mainly due to hydrogen cyanide. Appl Environ Microbiol 77:1000–1008. doi:10.1128/AEM.01968-10. 21115704 PMC3028696

[43] Peters BM, Jabra-Rizk MA, O’May GA, Costerton JW, Shirtliff ME. 2012. Polymicrobial interactions: impact on pathogenesis and human disease. Clin Microbiol Rev 25:193–213. doi:10.1128/CMR.00013-11. 22232376 PMC3255964

[44] Sanderson K, Wescombe L, Kirov SM, Champion A, Reid DW. 2008. Bacterial cyanogenesis occurs in the cystic fibrosis lung. Eur Respir J 32:329–333. doi:10.1183/09031936.00152407. 18480103

[45] Voggu L, Schlag S, Biswas R, Rosenstein R, Rausch C, Götz F. 2006. Microevolution of cytochrome bd oxidase in Staphylococci and its implication in resistance to respiratory toxins released by Pseudomonas. J Bacteriol 188:8079–8086. doi:10.1128/JB.00858-06. 17108291 PMC1698191

[46] Holloway BW. 1955. Genetic recombination in Pseudomonas aeruginosa. J Gen Microbiol 13:572–581. doi:10.1099/00221287-13-3-572. 13278508

[47] Castric KF, Castric PA. 1983. Method for rapid detection of cyanogenic bacteria. Appl Environ Microbiol 45:701–702. doi:10.1128/aem.45.2.701-702.1983. 16346217 PMC242347

[48] Kinkel TL, Roux CM, Dunman PM, Fang FC. 2013. The Staphylococcus aureus SrrAB two-component system promotes resistance to nitrosative stress and hypoxia. mBio 4:e00696-13. doi:10.1128/mBio.00696-13. 24222487 PMC3892780

[49] Stewart PS, Franklin MJ. 2008. Physiological heterogeneity in biofilms. Nat Rev Microbiol 6:199–210. doi:10.1038/nrmicro1838. 18264116

[50] Darch SE, Simoska O, Fitzpatrick M, Barraza JP, Stevenson KJ, Bonnecaze RT, Shear JB, Whiteley M. 2018. Spatial determinants of quorum signaling in a Pseudomonas aeruginosa infection model. Proc Natl Acad Sci USA 115:4779–4784. doi:10.1073/pnas.1719317115. 29666244 PMC5939081

[51] Smith H. 1982. The role of microbial interactions in infectious disease. Philos Trans R Soc Lond B Biol Sci 297:551–561. doi:10.1098/rstb.1982.0060. 6125962

[52] Sibley CD, Rabin H, Surette MG. 2006. Cystic fibrosis: a polymicrobial infectious disease. Future Microbiol 1:53–61. doi:10.2217/17460913.1.1.53. 17661685

[53] Ratjen F, Döring G. 2003. Cystic fibrosis. Lancet 361:681–689. doi:10.1016/S0140-6736(03)12567-6. 12606185

[54] Hubert D, Réglier-Poupet H, Sermet-Gaudelus I, Ferroni A, Le Bourgeois M, Burgel PR, Serreau R, Dusser D, Poyart C, Coste J. 2013. Association between Staphylococcus aureus alone or combined with Pseudomonas aeruginosa and the clinical condition of patients with cystic fibrosis. J Cyst Fibros 12:497–503. doi:10.1016/j.jcf.2012.12.003. 23291443

[55] Zolin A, Orenti A, van Rens J, Fox A, Krasnyk M, Jung A, Mei-Zahav M, Cosgriff R, Storms V, Naehrlich L. 2019. ECFSPR annual report 2017.

[56] Garcia-Medina R, Dunne WM, Singh PK, Brody SL. 2005. Pseudomonas aeruginosa acquires biofilm-like properties within airway epithelial cells. Infect Immun 73:8298–8305. doi:10.1128/IAI.73.12.8298-8305.2005. 16299327 PMC1307054

[57] Martin I, Waters V, Grasemann H. 2021. Approaches to targeting bacterial biofilms in cystic fibrosis airways. Int J Mol Sci 22:2155. doi:10.3390/ijms22042155. 33671516 PMC7926955

[58] Keller L, Surette MG. 2006. Communication in bacteria: an ecological and evolutionary perspective. Nat Rev Microbiol 4:249–258. doi:10.1038/nrmicro1383. 16501584

[59] O’Sullivan BP, Freedman SD. 2009. Cystic fibrosis. Lancet 373:1891–1904. doi:10.1016/S0140-6736(09)60327-5. 19403164

[60] Nguyen AT, Oglesby-Sherrouse AG. 2016. Interactions between Pseudomonas aeruginosa and Staphylococcus aureus during co-cultivations and polymicrobial infections. Appl Microbiol Biotechnol 100:6141–6148. doi:10.1007/s00253-016-7596-3. 27236810 PMC4916000

[61] Worlitzsch D, Tarran R, Ulrich M, Schwab U, Cekici A, Meyer KC, Birrer P, Bellon G, Berger J, Weiss T, Botzenhart K, Yankaskas JR, Randell S, Boucher RC, Döring G. 2002. Effects of reduced mucus oxygen concentration in airway Pseudomonas infections of cystic fibrosis patients. J Clin Invest 109:317–325. doi:10.1172/JCI13870. 11827991 PMC150856

[62] Ryall B, Davies JC, Wilson R, Shoemark A, Williams HD. 2008. Pseudomonas aeruginosa, cyanide accumulation and lung function in CF and non-CF bronchiectasis patients. Eur Respir J 32:740–747. doi:10.1183/09031936.00159607. 18480102

[63] Stutz MD, Gangell CL, Berry LJ, Garratt LW, Sheil B, Sly PD, Australian Respiratory Early Surveillance Team for Cystic Fibrosis. 2011. Cyanide in bronchoalveolar lavage is not diagnostic for Pseudomonas aeruginosa in children with cystic fibrosis. Eur Respir J 37:553–558. doi:10.1183/09031936.00024210. 20562125

[64] Gilchrist FJ, Bright-Thomas RJ, Jones AM, Smith D, Spaněl P, Webb AK, Lenney W. 2013. Hydrogen cyanide concentrations in the breath of adult cystic fibrosis patients with and without Pseudomonas aeruginosa infection. J Breath Res 7:e026010. doi:10.1088/1752-7155/7/2/026010. 23680696

[65] Yang L, Liu Y, Markussen T, Høiby N, Tolker-Nielsen T, Molin S. 2011. Pattern differentiation in co-culture biofilms formed by Staphylococcus aureus and Pseudomonas aeruginosa. FEMS Immunol Med Microbiol 62:339–347. doi:10.1111/j.1574-695X.2011.00820.x. 21595754

[66] Price CE, Brown DG, Limoli DH, Phelan VV, O’Toole GA. 2020. Exogenous alginate protects Staphylococcus aureus from killing by Pseudomonas aeruginosa. J Bacteriol 202:e00559-19. doi:10.1128/JB.00559-19. 31792010 PMC7099135

[67] Camus L, Briaud P, Vandenesch F, Moreau K. 2021. How bacterial adaptation to cystic fibrosis environment shapes interactions between Pseudomonas aeruginosa and Staphylococcus aureus. Front Microbiol 12:617784. doi:10.3389/fmicb.2021.617784. 33746915 PMC7966511

[68] Barraza JP, Whiteley M. 2021. A Pseudomonas aeruginosa antimicrobial affects the biogeography but not fitness of Staphylococcus aureus during coculture. mBio 12:e00047-21. doi:10.1128/mBio.00047-21. 33785630 PMC8092195

[69] Silva-Rocha R, Martínez-García E, Calles B, Chavarría M, Arce-Rodríguez A, de Las Heras A, Páez-Espino AD, Durante-Rodríguez G, Kim J, Nikel PI, Platero R, de Lorenzo V. 2013. The Standard European Vector Architecture (SEVA): a coherent platform for the analysis and deployment of complex prokaryotic phenotypes. Nucleic Acids Res 41:D666–D675. doi:10.1093/nar/gks1119. 23180763 PMC3531073

[70] Ghigo JM. 2001. Natural conjugative plasmids induce bacterial biofilm development. Nature 412:442–445. doi:10.1038/35086581. 11473319

[71] Davies DG, Parsek MR, Pearson JP, Iglewski BH, Costerton JW, Greenberg EP. 1998. The involvement of cell-to-cell signals in the development of a bacterial biofilm. Science 280:295–298. doi:10.1126/science.280.5361.295. 9535661

[72] Schroth MN, Cho JJ, Green SK, Kominos SD, Microbiology Society Publishing. 1977. Epidemiology of Pseudomonas aeruginosa in agricultural areas. J Med Microbiol 67:1191–1201. doi:10.1099/jmm.0.000758. 30067169

[73] Takeya K, Amako K. 1966. A rod-shaped Pseudomonas phage. Virology 28:163–165. doi:10.1016/0042-6822(66)90317-5. 4955194

[74] Khaledi A, Weimann A, Schniederjans M, Asgari E, Kuo TH, Oliver A, Cabot G, Kola A, Gastmeier P, Hogardt M, Jonas D, Mofrad MR, Bremges A, McHardy AC, Häussler S. 2020. Predicting antimicrobial resistance in Pseudomonas aeruginosa with machine learning-enabled molecular diagnostics. EMBO Mol Med 12:e10264. doi:10.15252/emmm.201910264. 32048461 PMC7059009

[75] Lebeaux D, Larroque B, Gellen-Dautremer J, Leflon-Guibout V, Dreyer C, Bialek S, Froissart A, Hentic O, Tessier C, Ruimy R, Pelletier AL, Crestani B, Fournier M, Papo T, Barry B, Zarrouk V, Fantin B. 2012. Clinical outcome after a totally implantable venous access port-related infection in cancer patients: a prospective study and review of the literature. Medicine (Baltimore) 91:309–318. doi:10.1097/MD.0b013e318275ffe1. 23117849

[76] Baba T, Takeuchi F, Kuroda M, Yuzawa H, Aoki K, Oguchi A, Nagai Y, Iwama N, Asano K, Naimi T, Kuroda H, Cui L, Yamamoto K, Hiramatsu K. 2002. Genome and virulence determinants of high virulence community-acquired MRSA. Lancet 359:1819–1827. doi:10.1016/s0140-6736(02)08713-5. 12044378

[77] Villanueva M, García B, Valle J, Rapún B, Ruiz de Los Mozos I, Solano C, Martí M, Penadés JR, Toledo-Arana A, Lasa I. 2018. Sensory deprivation in Staphylococcus aureus. Nat Commun 9:523. doi:10.1038/s41467-018-02949-y. 29410457 PMC5802764

[78] Valle J, Toledo-Arana A, Berasain C, Ghigo JM, Amorena B, Penadés JR, Lasa I. 2003. SarA and not sigmaB is essential for biofilm development by Staphylococcus aureus. Mol Microbiol 48:1075–1087. doi:10.1046/j.1365-2958.2003.03493.x. 12753197

[79] Shafer WM, Iandolo JJ. 1979. Genetics of staphylococcal enterotoxin B in methicillin-resistant isolates of Staphylococcus aureus. Infect Immun 25:902–911. doi:10.1128/iai.25.3.902-911.1979. 259057 PMC414533

[80] Duthie ES, Lorenz LL. 1952. Staphylococcal coagulase: mode of action and antigenicity. J Gen Microbiol 6:95–107. doi:10.1099/00221287-6-1-2-95. 14927856

[81] Herbert S, Ziebandt AK, Ohlsen K, Schäfer T, Hecker M, Albrecht D, Novick R, Götz F. 2010. Repair of global regulators in Staphylococcus aureus 8325 and comparative analysis with other clinical isolates. Infect Immun 78:2877–2889. doi:10.1128/iai.00088-10. 20212089 PMC2876537

[82] Kuroda M, Ohta T, Uchiyama I, Baba T, Yuzawa H, Kobayashi I, Cui L, Oguchi A, Aoki K, Nagai Y, Lian J, Ito T, Kanamori M, Matsumaru H, Maruyama A, Murakami H, Hosoyama A, Mizutani-Ui Y, Takahashi NK, Sawano T, Inoue R, Kaito C, Sekimizu K, Hirakawa H, Kuhara S, Goto S, Yabuzaki J, Kanehisa M, Yamashita A, Oshima K, Furuya K, Yoshino C, Shiba T, Hattori M, Ogasawara N, Hayashi H, Hiramatsu K. 2001. Whole genome sequencing of meticillin-resistant Staphylococcus aureus. Lancet 357:1225–1240. doi:10.1016/s0140-6736(00)04403-2. 11418146

[83] Kennedy AD, Otto M, Braughton KR, Whitney AR, Chen L, Mathema B, Mediavilla JR, Byrne KA, Parkins LD, Tenover FC, Kreiswirth BN, Musser JM, DeLeo FR. 2008. Epidemic community-associated methicillin-resistant Staphylococcus aureus: recent clonal expansion and diversification. Proc Natl Acad Sci USA 105:1327–1332. doi:10.1073/pnas.0710217105. 18216255 PMC2234137

[84] Ibberson CB, Parlet CP, Kwiecinski J, Crosby HA, Meyerholz DK, Horswill AR. 2016. Hyaluronan modulation impacts Staphylococcus aureus biofilm infection. Infect Immun 84:1917–1929. doi:10.1128/iai.01418-15. 27068096 PMC4907140

[85] Cucarella C, Solano C, Valle J, Amorena B, Lasa I, Penadés JR. 2001. Bap, a Staphylococcus aureus surface protein involved in biofilm formation. J Bacteriol 183:2888–2896. doi:10.1128/jb.183.9.2888-2896.2001. 11292810 PMC99507

