# Supplementary material for: Pseudomonas aeruginosa Production of Hydrogen Cyanide Leads to Airborne Control of Staphylococcus aureus Growth in Biofilm and In Vivo Lung Environments
Source: mBio. 2022 Sep 21;13(5):e02154-22. doi: 10.1128/mbio.02154-22 (PMC9600780; doi:10.1128/mbio.02154-22)
Supplement: FIG S1 [file mbio.02154-22-s0001.pdf]

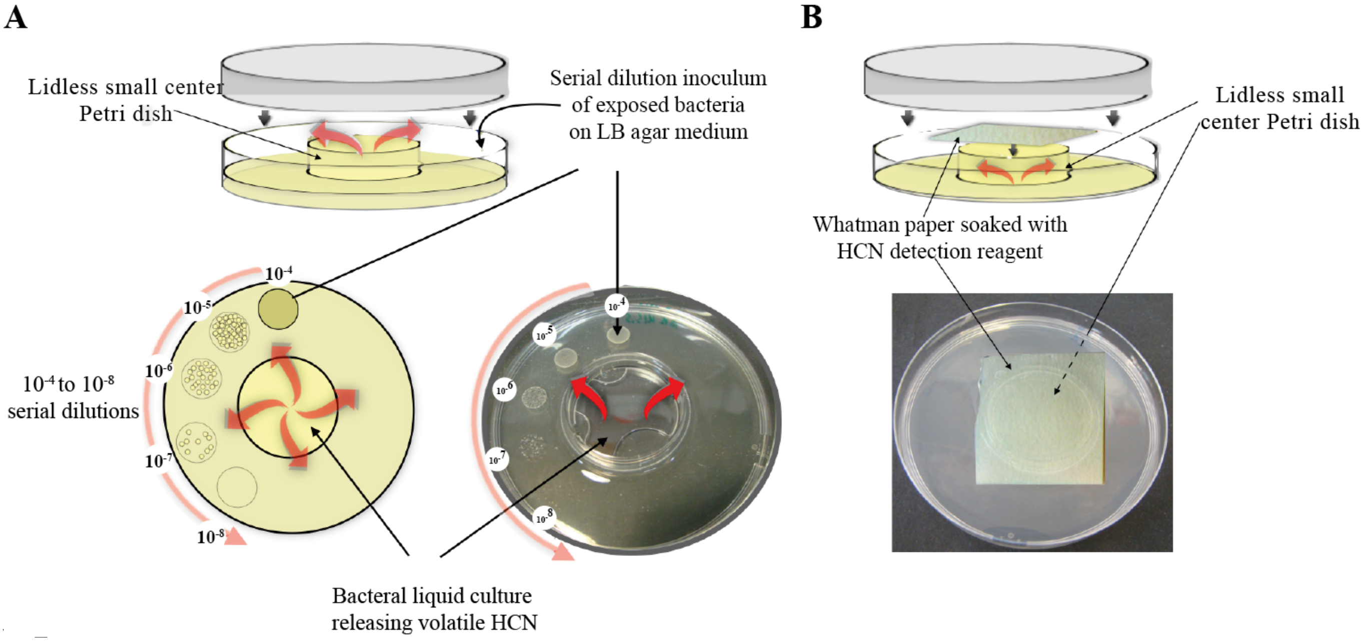

Supplementary Figure S1. **Two-Petri-dish assay**. **A:** Evaluation of volatile-mediated impact on growth between physically separated bacteria. A small lidless Petri dish is placed inside a larger one, which is closed by its lid. Serial dilutions of bacteria spotted on external LB agar ring are exposed to volatile molecule released from the culture placed in the central small Petri dish. Bacterial growth was monitored after 24h of incubation at 37°C, in aerobic or microaerobic conditions. **B:** Semi-quantitative HCN detection: Whatman chromatography paper soaked with HCN detection reagent was laid on the surface of the uncovered small Petri dish containing bacterial liquid culture releasing or not volatile HCN, and the large Petri dish was then closed and incubated for 24h at 37°C in aerobic or microaerobic conditions.
