# Supplementary material for: Pseudomonas aeruginosa Production of Hydrogen Cyanide Leads to Airborne Control of Staphylococcus aureus Growth in Biofilm and In Vivo Lung Environments
Source: mBio. 2022 Sep 21;13(5):e02154-22. doi: 10.1128/mbio.02154-22 (PMC9600780; doi:10.1128/mbio.02154-22)
Supplement: FIG S2 [file mbio.02154-22-s0002.pdf]

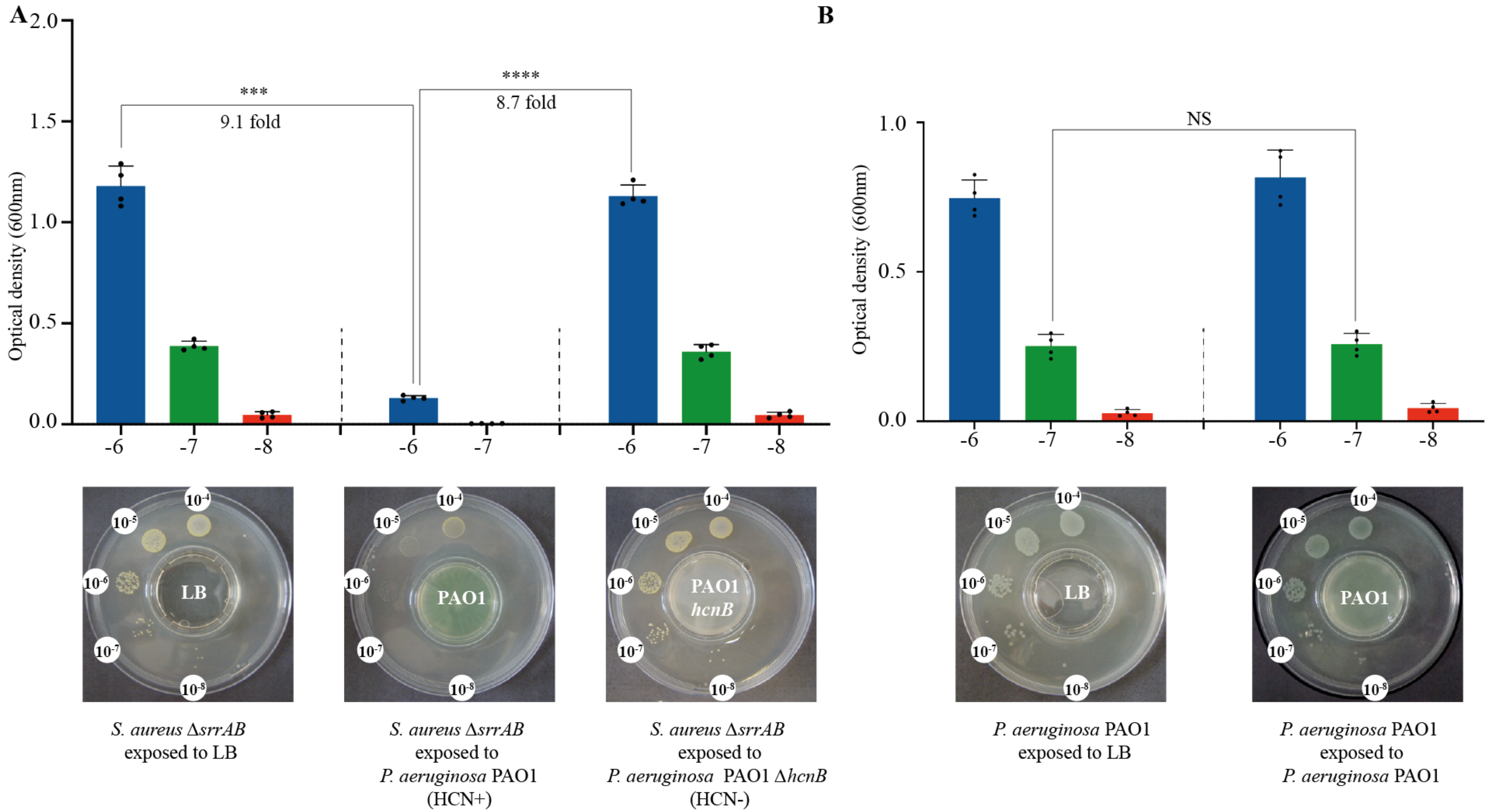

Supplementary Figure S2. ***P. aeruginosa* growth is not inhibited by HCN produced**. **A:** Control showing that HCN emitted by *P. aeruginosa* PAO1 inhibits *S. aureus* MW2 Δ*srrAB* mutant in aerobic conditions. **(top):** Graph representing the quantification of the effect of exposure of *S. aureus* Δ*srrAB* to WT or HCN-deficient *P. aeruginosa* PAO1 in LB aerobic conditions. Data correspond to the quantification

of the bacteria growing on 10^{-6} to 10^{-8} (respectively blue, green and red bars) dilution spots (see Fig. S1 for set up) exposed or not to *P. aeruginosa* HCN. Each spot was punched out from the LB agar plate, resuspended in PBS and the corresponding OD_{600nm} was determined. The fold differences observed between different conditions at comparable dilution are indicated. They were calculated based on the ratio of the mean of 4 independent quantifications at each dilution. **(bottom):** Serial dilution of *S. aureus* MW2Δ*srrAB* exposed to *P. aeruginosa* PAO1 WT or Δ*hcnB* cultures in LB in the 2-Petri-dish assay (see Supplementary Fig. S1). Each experiment was performed at least four times. **B:** Control showing that *P. aeruginosa* PAO1growth is insensitive to its own HCN. **(top):** same as A except that graph representing the quantification of the effect of exposure of *P. aeruginosa* PAO1 to WT or HCN-deficient *P. aeruginosa* PAO1 in LB aerobic conditions. **(bottom):** Serial dilution of *P. aeruginosa* PAO1 exposed to *P. aeruginosa* PAO1 WT or Δ*hcnB* cultures in LB in the 2-Petri-dish assay (see Supplementary Fig. S1). Each experiment was performed at least four times. Statistics correspond to two-tailed unpaired *t-test* with Welch correction. N.S.: not significant, *** p≤0.001 and **** p≤0.0001.
