# Supplementary material for: Pseudomonas aeruginosa Production of Hydrogen Cyanide Leads to Airborne Control of Staphylococcus aureus Growth in Biofilm and In Vivo Lung Environments
Source: mBio. 2022 Sep 21;13(5):e02154-22. doi: 10.1128/mbio.02154-22 (PMC9600780; doi:10.1128/mbio.02154-22)
Supplement: FIG S3 [file mbio.02154-22-s0003.pdf]

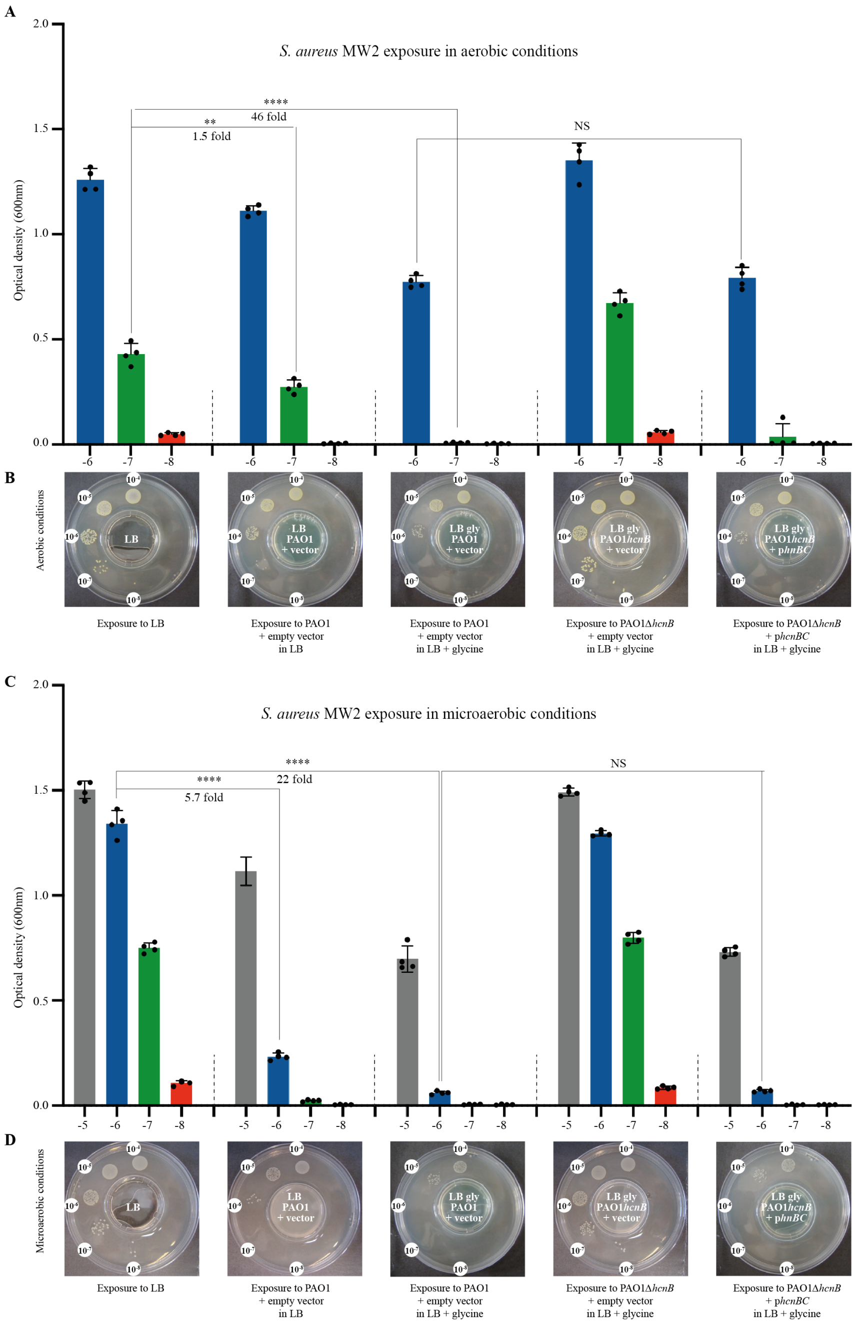

Supplementary Figure S3. **Complementation of the *hcnB* mutation in *P. aeruginosa* PAO1**. **A:** Graph representing the quantification of the effect of exposure of *S. aureus* MW2 to *P. aeruginosa* PAO1 WT or Δ*hcnB* mutant either carrying the empty vector pSEVA238 or p*hcnBC* in LB + the inducer sodium benzoate (2 mM) in aerobic conditions. Data correspond to the quantification of the bacteria growing on 10^{-5} to 10^{-8} (respectively grey, blue, green and red bars) dilution spots (see Fig. S1 for set up) exposed or not to *P. aeruginosa* HCN. Each spot was punched out from the LB agar plate, resuspended in PBS and the corresponding OD_{600nm} was determined. The fold differences observed between different conditions at comparable dilution are indicated. They were calculated based on the ratio of the mean of 4 independent quantifications at each dilution.

B: Serial dilution of *S. aureus* MW2 exposed to *P. aeruginosa* WT or Δ*hcnB* cultures in LB supplemented or not with 0.4% (w/v) glycine in the 2-Petri-dish assay in aerobic conditions (see Supplementary Fig. S1). Pictures were taken after 24h of incubation at 37°C in aerobic conditions. Each experiment was performed at least three times. **C** and **D:** same as A and B except that the experiments were performed in microaerobic conditions. Statistics correspond to two-tailed unpaired *t-test* with Welch correction. N.S.: not significant, ** $p \leq 0.01$ and **** $p \leq 0.0001$.
