# Supplementary material for: Pseudomonas aeruginosa Production of Hydrogen Cyanide Leads to Airborne Control of Staphylococcus aureus Growth in Biofilm and In Vivo Lung Environments
Source: mBio. 2022 Sep 21;13(5):e02154-22. doi: 10.1128/mbio.02154-22 (PMC9600780; doi:10.1128/mbio.02154-22)
Supplement: FIG S4 [file mbio.02154-22-s0004.pdf]

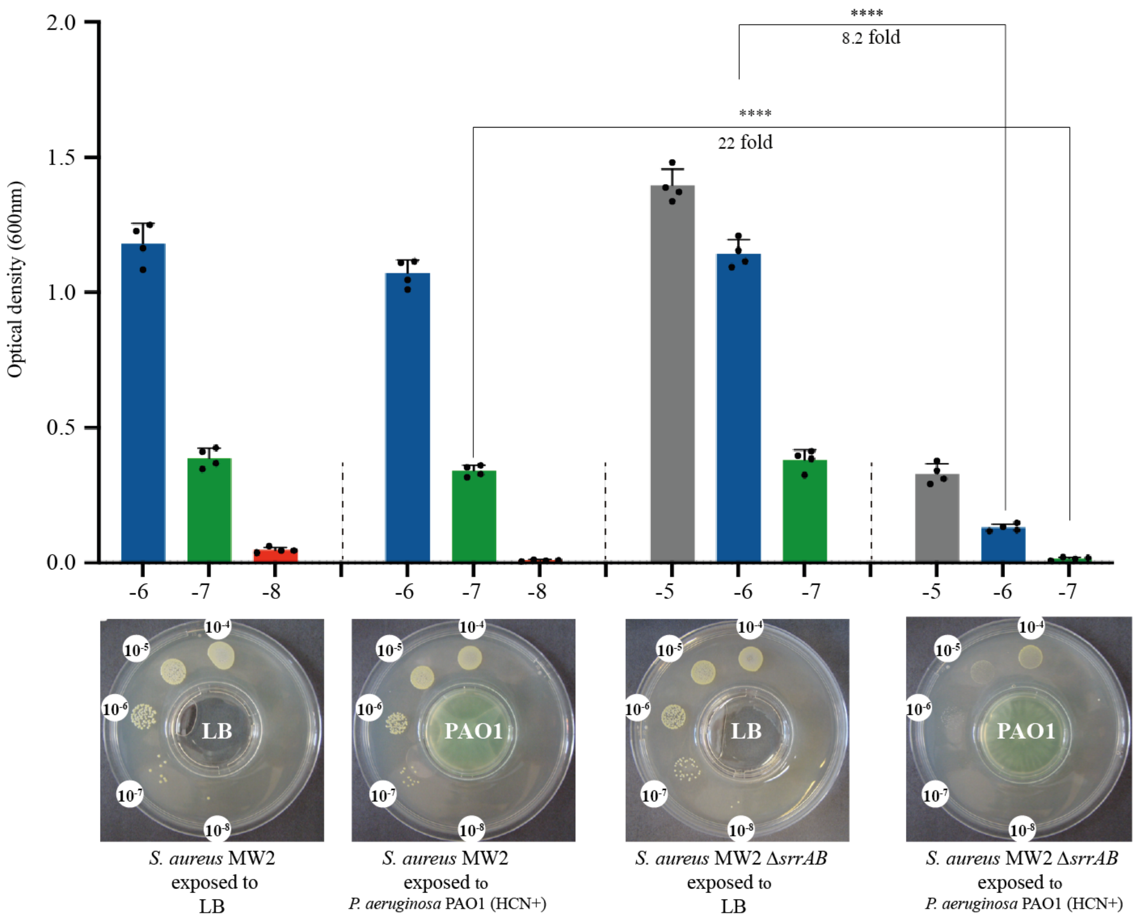

Supplementary Figure S4. ***S. aureus* MW2 *srrAB* mutant displayed increased sensitivity to HCN**. **Top:** Graph representing the quantification of the effect of exposure of *S. aureus* MW2 or to MW2Δ*srrAB* mutant to HCN produced by *P. aeruginosa* WT in LB under aerobic conditions. Data correspond to the quantification of the bacteria growing on 10^{-6} to 10^{-8} (respectively blue, green and red bars) dilution spots (see Fig. S1 for set up) exposed or not to *P. aeruginosa* HCN. Each spot was punched out from the LB agar plate, resuspended in PBS and the corresponding OD_{600nm} was determined. The fold differences observed between different conditions at comparable dilution are indicated. They were calculated based on the ratio of the mean of 4 independent quantifications at each dilution. **Bottom:** Serial dilution of *S. aureus* MW2 or MW2Δ*srrAB* exposed to HCN produced by *P. aeruginosa* WT in LB in aerobic conditions in the 2-Petri-dish assay (see Supplementary Fig. S1). Pictures were taken after 24h of incubation at 37°C in aerobic conditions. Statistics correspond to two-tailed unpaired *t-test* with Welch correction. Each experiment was performed at least four times. **** $p \leq 0.0001$
