# Supplementary material for: Pseudomonas aeruginosa Production of Hydrogen Cyanide Leads to Airborne Control of Staphylococcus aureus Growth in Biofilm and In Vivo Lung Environments
Source: mBio. 2022 Sep 21;13(5):e02154-22. doi: 10.1128/mbio.02154-22 (PMC9600780; doi:10.1128/mbio.02154-22)
Supplement: FIG S5 [file mbio.02154-22-s0005.pdf]

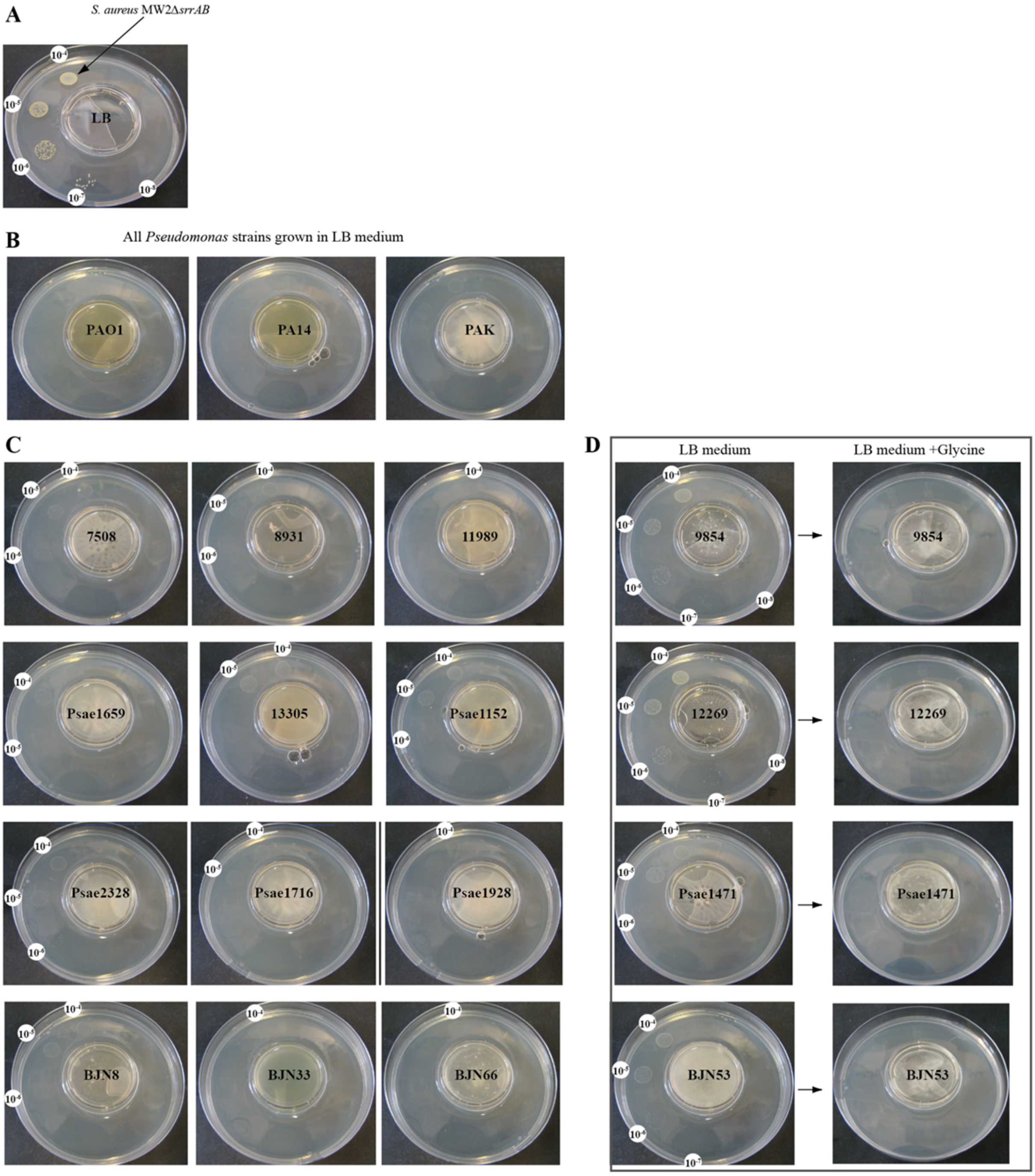

Supplementary figure S5. **Production of HCN is widespread among *Pseudomonas aeruginosa* strains and clinical isolates**. **A:** Growth of serial dilutions of *S. aureus* MW2 *srrAB* mutant upon exposure to LB (**A**) or to a panel of laboratory (**B**) and clinical (**C**) *P. aeruginosa* strains grown in LB after 24h incubation at 37°C in aerobic conditions, using the 2-petri-dish assay described in Fig S1. **D:** For strains that only partially showed reduced *S. aureus* growth, 0.4% (w/v) glycine was also added in LB medium. Each experiment was performed at least three times. The dilutions were indicated only on the visible growth spots.
