# Supplementary material for: Pseudomonas aeruginosa Production of Hydrogen Cyanide Leads to Airborne Control of Staphylococcus aureus Growth in Biofilm and In Vivo Lung Environments
Source: mBio. 2022 Sep 21;13(5):e02154-22. doi: 10.1128/mbio.02154-22 (PMC9600780; doi:10.1128/mbio.02154-22)
Supplement: FIG S6 [file mbio.02154-22-s0006.pdf]

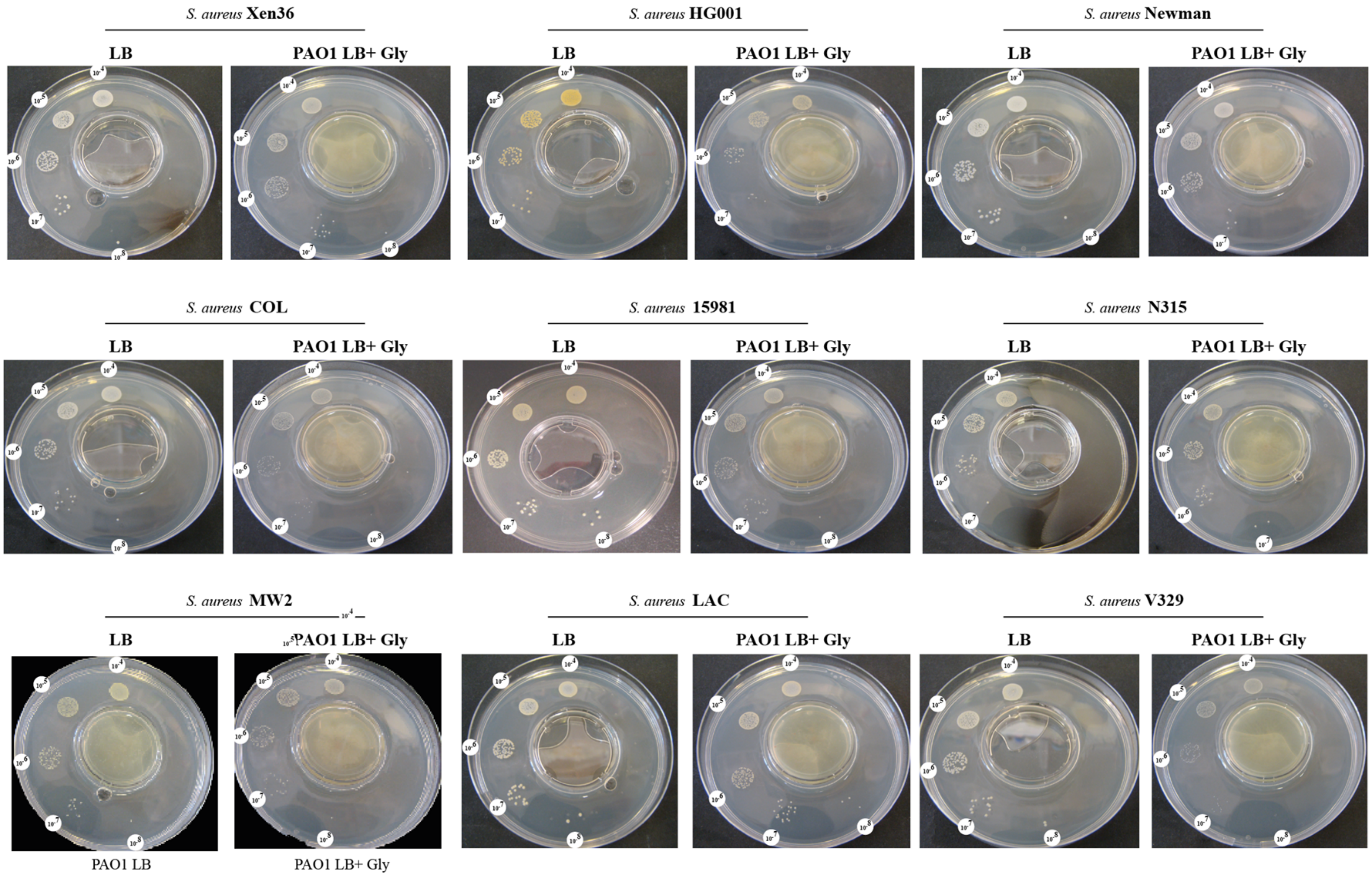
S. aureus Xen36
LB
PAO1 LB+ Gly
S. aureus HG001
LB
PAO1 LB+ Gly
S. aureus Newman
LB
PAO1 LB+ Gly
S. aureus COL
LB
PAO1 LB+ Gly
S. aureus 15981
LB
PAO1 LB+ Gly
S. aureus N315
LB
PAO1 LB+ Gly
S. aureus MW2
LB
PAO1 LB+ Gly
S. aureus LAC
LB
PAO1 LB+ Gly
S. aureus V329
LB
PAO1 LB+ Gly
PAO1 LB
PAO1 LB+ Gly

Supplementary figure S6. **Growth inhibition of a panel of *S. aureus* strains upon aerial exposure to *P. aeruginosa* PAO1 culture**. The growth of serial dilution of a panel of *S. aureus* strains upon exposure to *P. aeruginosa* PAO1 cultures in LB supplemented with 0.4% (w/v) glycine after 24h incubation at 37°C in aerobic conditions, using the 2-petri-dish assay as described in Fig S1. Each experiment was performed at least three times.
