# Supplementary material for: Pseudomonas aeruginosa Production of Hydrogen Cyanide Leads to Airborne Control of Staphylococcus aureus Growth in Biofilm and In Vivo Lung Environments
Source: mBio. 2022 Sep 21;13(5):e02154-22. doi: 10.1128/mbio.02154-22 (PMC9600780; doi:10.1128/mbio.02154-22)
Supplement: FIG S7 [file mbio.02154-22-s0007.pdf]

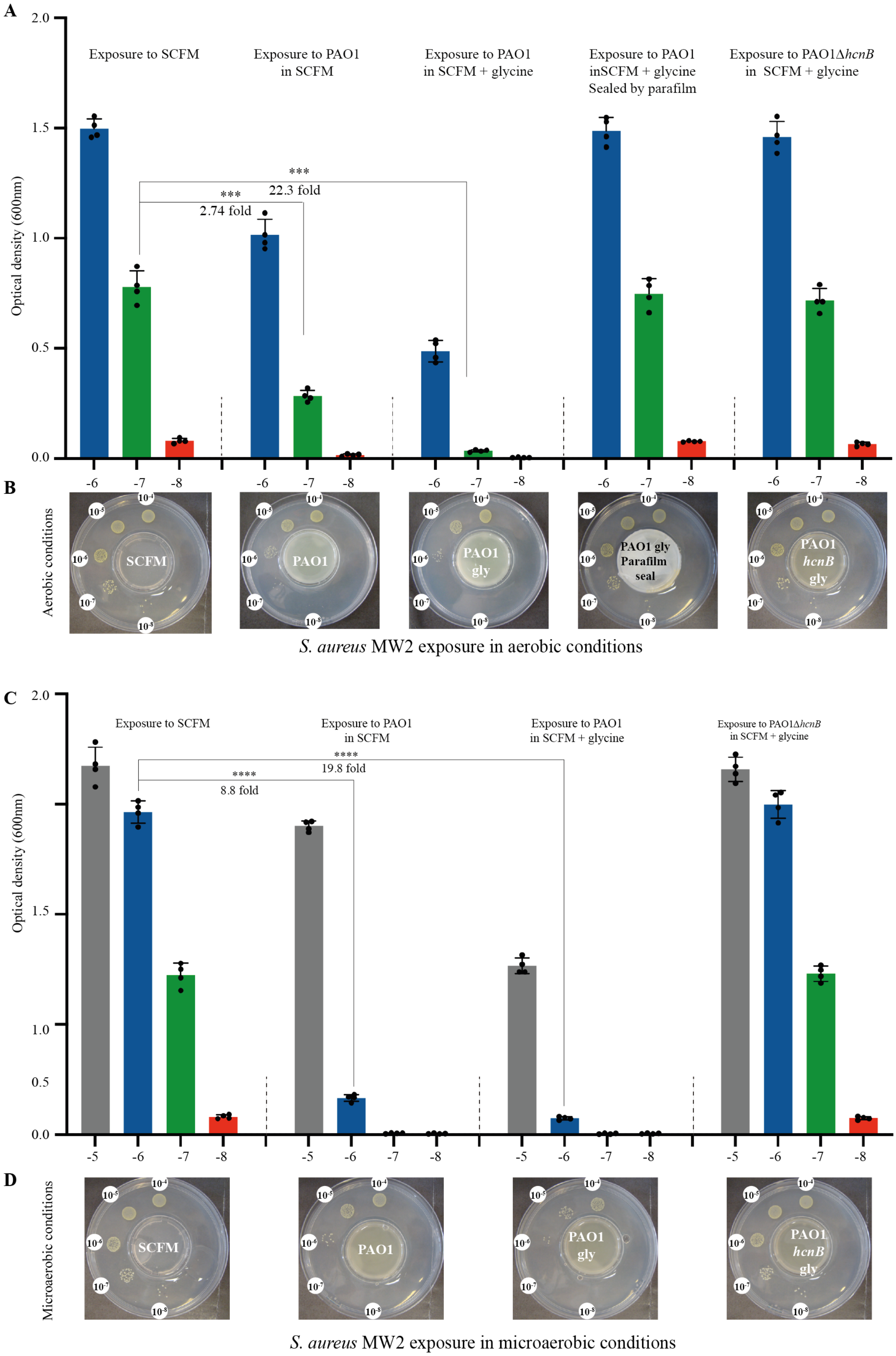
A
B
Optical density (600nm)
Exposure to SCFM
Exposure to PAO1 in SCFM
Exposure to PAO1 in SCFM + glycine
Exposure to PAO1 inSCFM + glycine Sealed by parafilm
Exposure to PAO1ΔhcnB in SCFM + glycine
*** 2.74 fold
*** 22.3 fold
Aerobic conditions
SCFM
PAO1
PAO1 gly
PAO1 gly Parafilm seal
PAO1 hcnB gly
S. aureus MW2 exposure in aerobic conditions
C
D
Exposure to PAO1 in SCFM + glycine
Exposure to PAO1ΔhcnB in SCFM + glycine
**** 8.8 fold
**** 19.8 fold
Microaerobic conditions
S. aureus MW2 exposure in microaerobic conditions

1
2

Supplementary Figure S7. ***P. aeruginosa*** **production of HCN in SCFM2 medium also leads to airborne inhibition of** ***S. aureus*** **growth**.

A: Graph representing the quantification of the effect of exposure to *P. aeruginosa* HCN on *S. aureus* MW2 growth in SCFM2 aerobic conditions. Data correspond to the quantification of the bacteria growing on 10^{-5} to 10^{-8} (respectively grey, blue, green and red bars) dilution spots (see Fig. S1 for set up) and exposed or not to *P. aeruginosa* HCN. Each spot was punched out from the LB agar plate, resuspended in PBS and the corresponding OD_{600nm} was determined. The fold differences observed between different conditions at comparable dilution are indicated. They were calculated based on the ratio of the mean of 4 independent quantifications at each dilution. **B:** Serial dilution of *S. aureus* MW2 exposed to *P. aeruginosa* WT or Δ*hcnB* cultures in SCFM2 supplemented or not with 0.4% (w/v) glycine in the 2-Petri-dish assay (see Supplementary Fig. S1). No inhibition of *S. aureus* MW2 growth is observed when the middle small Petri dish containing *P. aeruginosa* culture is sealed with parafilm. Pictures were taken after 24h of incubation at 37°C in aerobic conditions. Each experiment was performed at least three times. **C** and **D** as in A and B except that the experiments were performed in microaerobic conditions. Statistics correspond to two-tailed unpaired *t-test* with Welch correction. *** $p \leq 0.001$ and **** $p \leq 0.0001$.
